# An iterative approach for estimating domain-specific cognitive abilities from large scale online cognitive data

**DOI:** 10.1038/s41746-024-01327-x

**Published:** 2024-11-19

**Authors:** Valentina Giunchiglia, Dragos-Cristian Gruia, Annalaura Lerede, William Trender, Peter Hellyer, Adam Hampshire

**Affiliations:** 1 Department of Brain Sciences, Imperial College London, London, UK; 2 Centre for Neuroimaging Sciences, Institute of Psychiatry, Psychology & Neuroscience, King’s College London, London, UK; 3 Department of Biomedical Informatics, Harvard University, Boston, USA; 4 Present Address: Centre for Neuroimaging Sciences, Institute of Psychiatry, Psychology & Neuroscience, King’s College London, London, UK

**Keywords:** Human behaviour, Cognitive neuroscience, Computational neuroscience

## Abstract

Online cognitive tasks are gaining traction as scalable and cost-effective alternatives to traditional supervised assessments. However, variability in peoples’ home devices, visual and motor abilities, and speed-accuracy biases confound the specificity with which online tasks can measure cognitive abilities. To address these limitations, we developed IDoCT (Iterative Decomposition of Cognitive Tasks), a method for estimating domain-specific cognitive abilities and trial-difficulty scales from task performance timecourses in a data-driven manner while accounting for device and visuomotor latencies, unspecific cognitive processes and speed-accuracy trade-offs. IDoCT can operate with any computerised task where cognitive difficulty varies across trials. Using data from 388,757 adults, we show that IDoCT successfully dissociates cognitive abilities from these confounding factors. The resultant cognitive scores exhibit stronger dissociation of psychometric factors, improved cross-participants distributions, and meaningful demographic’s associations. We propose that IDoCT can enhance the precision of online cognitive assessments, especially in large scale clinical and research applications.

## Introduction

Automated online assessment technology is rapidly gaining popularity as an alternative to traditional supervised tests for measuring cognitive abilities, with applications across diverse contexts, including fundamental research into human intelligence and cognitive processes^1^, evaluating cognitive differences in epidemiological cohorts and registers^2,3^, assessing outcomes in clinical trials^4^ and providing diagnostics in healthcare^5^. This popularity is motivated by practical advantages; most notably, remote deployment via the devices participants already own under unsupervised conditions enables very large-scale population data to be collected, including across multiple timepoints, at a fraction of the cost of standard supervised assessment methods^6^. When designed and deployed carefully, online assessment technology is not just a convenient alternative to popular neuropsychological scales, it can also surpass them in sensitivity and specificity^7–10^.

Nonetheless, online assessment is not without limitations. Most notably, people own a continuously changing zoo of computer, smartphone and tablet devices that vary in screen size and response interface. Differences in device configurations are not reliably measurable from within a browser, which is problematic as they can confound the accuracy and response time (RT) recordings that are fundamental to objective cognitive assessment. In addition, it is well established that cognitive tasks do not measure individual abilities in isolation, even when deployed on the same device under controlled conditions. Instead, all tasks involve multiple underlying processes, but combined in different mixtures. For example, computerised tasks designed to measure spatial planning abilities also depend on perceptual processing and motor dexterity. This mixing of processes complicates the interpretability of summary score metrics from individual tasks, especially in clinical conditions that are characterised by concomitant cognitive and visuomotor deficits such as Parkinson’s disease, stroke or multiple sclerosis^11^. Careful design guided by fundamental experimental psychological principles can mitigate the sensitivity of tasks to the confounding effects of device, mild visuomotor abilities and cognitive processes they are not intended to measure^12^; however, these confounds are seldom entirely accounted for.

A potential solution to this problem is provided by the fact that, unlike classic pen-and-paper assessments, computerised tasks precisely record the timing and stimulus type for each response, providing a detailed trial-by-trial timecourse of each participant’s performance. Common approaches to summarise participant performance of a task tend not to fully utilise these detailed timecourses, relying instead on simple averaging or contrasting of accuracies and response speeds across conditions. The resultant summary scores do not fully resolve the above issues and are further complicated by speed-accuracy trade-offs (i.e., trade-off between replying slower and making more errors), which are known to bias specificity and sensitivity in a wide range of tasks^13^. Although some summary methods attempt to handle the speed-accuracy trade-off (e.g., inverse efficiency score - IES^14^, linear integrated speed – accuracy score - LISAS^15^ and balanced integration score - BIS^16^), they do not address the issue of multiple factors (i.e., visual, motor, device and task-specific and unspecific cognitive) contributing to performance.

Modelling approaches can potentially leverage trial-by-trial data to extract more precise measures of abilities. For example, drift diffusion models (DDMs) are designed to cognitive dissociate decision times from other confounding processes. A limitation is that DDMs were designed to model behaviour on simple binary choice tasks; generalisation to more complex cases, e.g., with multiple difficulty parameters or decision stages and more available response-choices^17–19^, requires careful adaptations and customisation for each task. Furthermore, even simple formulations of DDMs are computationally expensive, which is sub-optimal when handling very large online data, and integrating task complexities significantly increase these computational demands^18^. A more flexible approach is multiple item response theory, where differences in individual ‘items’ (or trials) are evaluated in terms of how well they can discriminate participants of different abilities^20^, including in applications where multiple latent abilities contribute to performance^21,22^. The principles of multiple item response theory can potentially be extended to account for the impact of external factors on performance, such as the device a person is tested on. Furthermore, although primarily focused on response accuracy, it is possible to include measures of response latency, enabling speed-accuracy trade-offs to be accounted for. A modelling approach that leverages these properties could produce more precise online assessment of specific cognitive abilities.

**Fig. 1:**
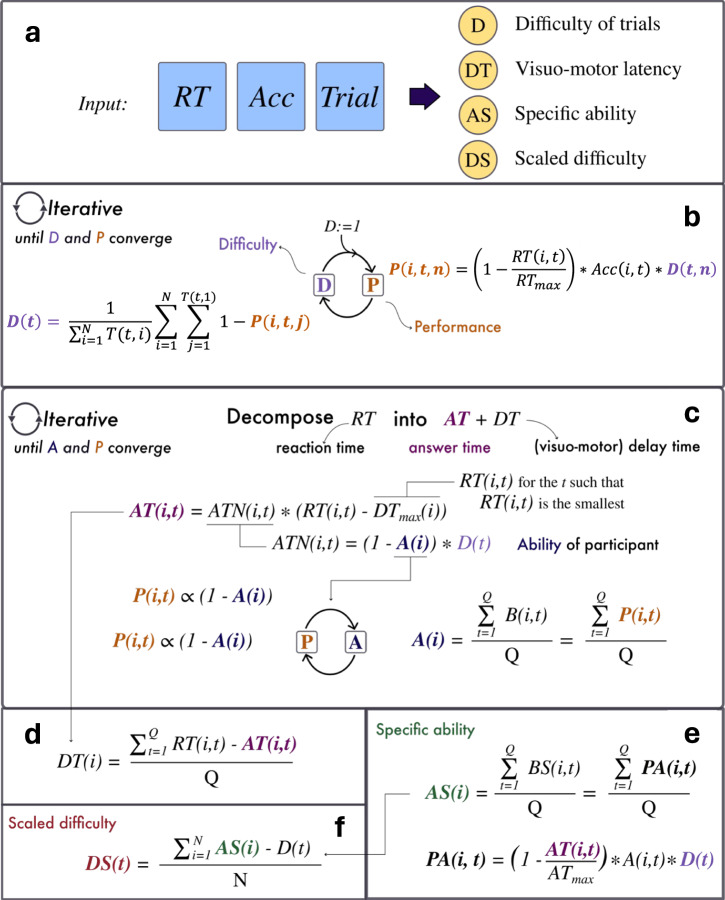
IDoCT model summary. IDoCT provides measures of cognitive ability (AS), delay time (DT), scaled (DS) and unscaled (D) trial-difficulty scales by parsing timecourses comprising trial-by-trial accuracy, RT and trials’ descriptive labels from a population sample (**a**). This is achieved in a series of steps. First, an unscaled measure of trial’s difficulty D is obtained through an iterative process that relies on the assumption that the difficulty of each trial (i.e., D) can be defined from the group’s performance (i.e., P) on that trial, which in turn depends both on the RT and accuracy. This process ends when the trial difficulty and the group’s performance converge on the same values (**b**). Then, a second iterative process is performed, where the previously obtained values of D are used to estimate a measure of answer time AT. This second iteration assumes that the RT comprises both the answer time AT (i.e., time required to complete the cognitive task) and delay time DT (i.e., delay time due to visuomotor, unspecific cognitive processes and device latency). The AT depends on the difficulty of the trials, as well as the ability of participants (i.e., A), which is defined as the cumulative performance P across all trials. An iterative process is obtained, since the measure of ability A depends on the performance P, which in turn depends on AT that is derived from A. The iterative process ends when the measures of ability and performance converge (**c**). Once AT is calculated, the delay time DT can be obtained as the average difference between RT and AT across all trials (**d**). At the same time, a measure of ability AS is calculated as the cumulative specific performance (i.e., PA) obtained by considering only the portion of RT that belongs to the cognitive process (i.e., AT) (**e**). Finally, a measure of scaled difficulty is computed, where the difficulty D of the trials is scaled according to the ability of the participants that completed those trials. This allows to inspect how the difficulty varies, at the group level, when the task design is characterised by a biased sampling approach (**f**).

This is why we developed and validated IDoCT (Iterative Decomposition of Cognitive Tasks), a novel iterative modelling approach that builds on principles of item theory in order to address all of the above-mentioned limitations in a computationally-efficient manner that is suitable for very large online datasets, while being flexible enough to be applied to virtually any computerised assessment task that manipulates cognitive difficulty across trials (See Fig. 1). IDoCT uses a fixed-point iterative process that handles the circularity of simultaneously defining individual abilities from trial difficulties and trial difficulties from individual performance. On the one hand, it works to assess a person’s ability to manage targeted cognitive demands (i.e., Specific Ability AS), while simultaneously handling the speed accuracy trade-off and modelling the confounding factors related to device latencies, visuomotor ability and cognitive abilities that are non-task specific (i.e., Delay Time DT - which cannot be attributed to trial-by-trial variability in the identified task dimensions). Critically, this is achieved without the need for explicit device labels, thereby not requiring ongoing updates to normative datasets over time. On the other hand, it recalculates the relative difficulty assigned to each trial (i.e., Difficulty D) across the task space based on the mass performance of all participants, and by using a scaling approach that accounts for the problem of bias in sampling of more difficult trials in higher performing individuals, as is commonly the case for adaptive computerised tasks (i.e., Difficulty Scaled DS). This results in a robust data-driven estimation of cognitive abilities and data-driven calibration of trial-difficulty scales.

To validate IDoCT, we apply it to data from a large cohort comprising 388,757 adults, across 12 different computerised tasks from the Cognitron platform^7,9,23^, which delivers tests of memory, reasoning, executive function, language and motor speed via home computer, tablet and smartphone devices. First, we confirm that IDoCT can accurately estimate cognitive abilities, visuomotor response speeds and trial difficulty from simulated data, where the ground truth is known, under conditions with and without bias due to adaptive difficulty routines. Then, we determine whether IDoCT produces difficulty scales that map onto the aspects of cognitive demand that each Cognitron task is intended to measure when applied to real data. Next, we examine participants’ IDoCT ability estimates and contrast them to raw scores in terms of form, device sensitivity and strength of association with demographic factors. Finally, we model the latent variable structure of the cognitive and visuomotor ability estimates to determine whether (a) they cluster in a manner that relates to cognitive domain and visuomotor demands respectively, (b) the task-latent variable mappings are more discrete than raw score metrics and (c) the component of variance explained by an overarching ‘g’ factor is minimised insofar as is possible to facilitate multivariate profiling of different cognitive abilities.

## Results

### Online dataset

**Fig. 2:**
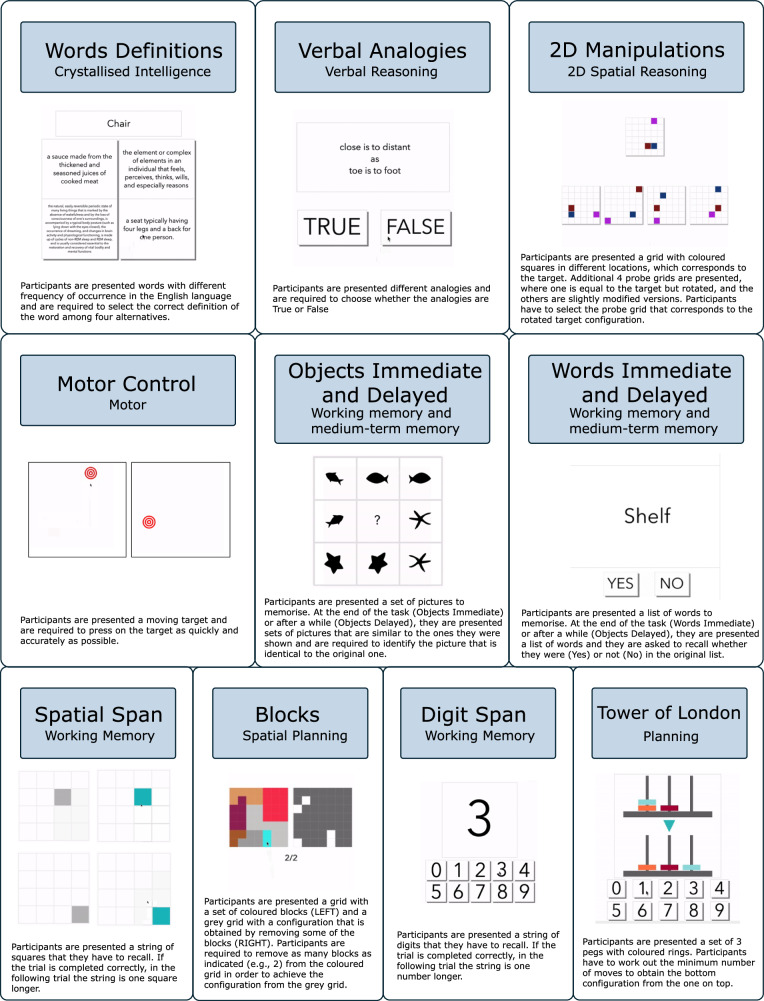
Summary of task designs. 12 tasks were included in the study: Blocks (spatial planning), Digits Span (working memory), Tower of London—TOL (planning), Spatial Span (working memory), Words Immediate and Delayed (recognition memory), Objects Immediate and Delayed (recognition memory), Motor Control (motor and attention), Verbal Analogies (semantic and verbal reasoning), Words Definition (crystallised intelligence) and 2D Manipulations (visuospatial reasoning).

Performance data were analysed for 388,757 individuals, who completed at least one of the 12 selected tasks from the Cognitron website^7,24^ between 2019 and 2022, and to simulated datasets, where the ground truth is known. Simulations are presented in Supplementary Fig. 1. Fig. 2 summarises designs of the online cognitive tasks. In brief, the tasks were designed to measure different aspects of memory, attention, reasoning and planning. They were brief, engaging and minimally gamified to improve compliance and completion rates when delivered online. On-the-fly algorithms for generating novel problems along balanced difficulty dimensions were implemented to minimise learning across repeat assessments. They had been optimised based on piloting with patients and older adults to ensure accessibility whilst maintaining sensitivity to cognitive deficits, minimise sensitivity to device and decorrelate across cognitive domains to reduce loading on a global ‘g’ factor^25,26^.

On average, participants completed 7.3 tasks, with 29,700 completing all 12. Overall demographics for the sample are presented in Table 1 and the number of times each task was completed is reported in Table 2. Since the model is applied on each task independently, no missing data imputation was required for the training of the model, and sparse data could be used. In brief, the cohort consisted of predominantly native English speakers (88.52%) living within the UK (85.13%) from a broad age range with approximately equal male-female split and bias toward higher education (57.34% with a degree), but inclusive of thousands of non-binary individuals, >14% non-white and a wide variety of work and economic status.

**Table 1:** Participants’ demographics

	Features	Percentage (%)
Education	Pre-GCSE	2.67
	School	3.19
	Degree	57.34
	PhD	4.81
	Missing	3.19
Occupation	Worker	65.55
	Retired	14.43
	Student	9.09
	Unemployed	3.00
	Homemaker	3.00
	Sheltered Employment	1.04
	Unknown	0.63
	Missing	3.23
Sex	Female	45.89
	Male	52.37
	Other	1.03
	Missing	0.70
Ethnicity	White	84.02
	Asian or Asian British	8.25
	Mixed or multiple ethnic groups	2.79
	American Hispanic	2.25
	Black, Black British, Caribbean or African	1.00
	Other ethnic groups	0.02
	Unknown	1.48
	Missing	0.15
Residence	United Kingdom	85.13
	Other	14.16
	Missing	0.71
Language	English	88.52
	Other	10.77
	Missing	0.70

The demographics are reported in percentages (%).

**Table 2:** Summary measures of D, DS, AS and DT

Task	D (mean ± sd)	DS (mean ± sd)	AS (mean ± sd)	DT (mean ± sd)	Number of participants
Motor Control	0.64±0.01	0.63±0.01	0.57±0.05	609±176	32,752
Blocks	0.64.±0.05	0.63±0.05	0.42±0.12	3602±1162	356,150
Digit Span	0.72±0.10	0.75±0.12	0.35±0.06	759±212	36,392
Spatial Span	0.78±0.15	0.74±0.13	0.34±0.06	689±209	357,770
2D Manipulations	0.59±0.05	0.59±0.05	0.49±0.06	4238±1371	359,185
TOL	0.64±0.05	0.64±0.05	0.38±0.16	8948±2774	356,948
Object memory delayed	0.63±0.01	0.63±0.01	0.54±0.06	2418±552	53,561
Object memory immediate	0.63±0.01	0.63±0.01	0.51±0.06	3024±700	56,320
Words memory delayed	0.62±0.04	0.62±0.04	0.49±0.06	846±139	104,921
Words memory immediate	0.61±0.02	0.61±0.02	0.52±0.05	887±135	105,592
Verbal Analogies	0.58±0.09	0.59±0.09	0.45±0.07	3194±855	346,356
Words definition	0.66±0.08	0.67±0.08	0.44±0.09	4621±1310	358,894

The table reports the mean ± standard deviation of the estimated measures of AS, DT, D and DS, as well as the number of participants that were included in the analysis of each cognitive task.

### Measures of trials’ difficulty D and DS give insights into the most challenging aspects of each task

**Fig. 3:**
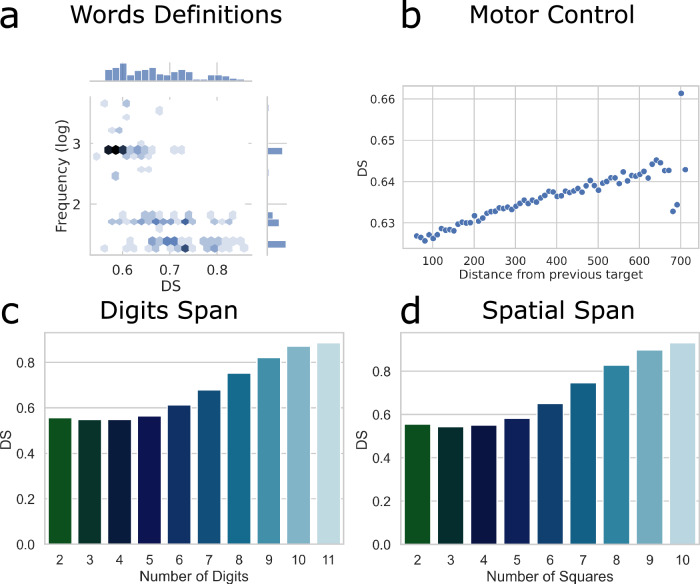
Estimated trial difficulty measure DS for Words Definitions, Motor Control, Digits Span and Spatial Span. **a** Words definitions: most frequently used words in the English language are associated with easier trials. **b** Motor Control: the further the target from the previous location, the more difficult the trial. **c** Digits Span: the more digits participants have to remember, the higher the DS score. **d** Spatial Span: the more squares participants have to remember, the higher the DS score.

IDoCT was applied separately to each cognitive task analysed in the study. The first step of the model consisted in the computation of trials’ difficulty D. IDoCT converged after ten iterations for all cognitive tasks, where the mean change in difficulty D tended to 0 across all types of trials (see Supplementary Fig. 2). The model tries to find estimates of trial difficulty that explain observed variability in performance (represented as a combination of accuracy and RT) across participants and trials. The difficulty estimates stop changing across iterations when the model derives difficulty measures that best explain the observed data. Concretely, further iterations could be computed, and the improvement would be negligible (i.e., tend to zero). The trial difficulty results are summarised in Fig. 3 and Table 2, where the mean difficulty D and DS per task is provided. Full results are available in Supplementary Figures 3 and 4.

For IDoCT to run, the individual trials of a task must be labelled according to differences in modulated difficulty parameters, which are idiosyncratic given what a task is designed to measure, e.g., number of moves, spatial distance or presented word, interstimulus interval, etc. The trial difficulty estimates (DS and D) and measures of individuals’ cognitive abilities (AS) relate to the specific demands that a given task manipulates. Fig. 3 reports how DS estimates, based on the overall populations’ performances, vary with trial demands in the cases of Digits Span, Spatial Span, Words Definitions and Motor control. Detailed analysis for all tasks is reported in Supplementary Figs. 3 and 4, where the interpretation of the derived DS is presented for each task.

Digits Span is a cognitive task designed to measure working memory capacity. Participants are presented with a sequence of numbers that they must recall. The first sequence is always 2 numbers long, and at each trial, if the participant replies correctly, the length of the next sequence increases by one. If the participant incorrectly recalls the sequence of numbers, then another string of the same length is presented. This is repeated until the participant replies incorrectly 3 times consecutively, when the task terminates. Spatial Span has a similar design to Digits Span, but instead of digits participants recall the location of sequences of flashes on a 4 × 4 grid of squares, testing their spatial working memory capacity. As shown in Fig. 3c for Digits Span, the derived DS measure suggested that the more numbers participants must remember, the harder is the trial (correlation between sequence length and DS is *r* = 0.92). Similarly for Spatial Span, the more squares participants have to remember, the harder is the trial (correlation between sequence length and DS is *r* = 0.84) (Fig. 3d).

Words Definitions is designed to measure crystallised intelligence. Participants are presented with a word and four alternative definitions. They must select the definition that corresponds to the meaning of the word as quickly as possible. The task has multiple trials and at each trial a different word is shown. The words are characterised by different frequency of occurrence in the English language. To assess the word difficulty scale predicted by IDoCT for the Words Definitions task, the correlation between the obtained measure of difficulty DS and the frequency of occurrence in the English language of the words included in the cognitive assessment was calculated. As shown in Fig. 3a, the most frequently used words in English were assigned a lower D and DS compared to the least frequently used ones. A Spearman correlation of −0.66 and −0.64 between respectively DS and D with the log frequency measures of the words was found.

Motor Control is designed to measure motor abilities. Participants are shown a moving target, which is represented as a circular red button, and are expected to press on the target as quickly as possible, as soon as it appears, and before it changes location in the screen. According to IDoCT, the further the location of the current target from the previous location, the higher the difficulty estimate DS (Spearman correlation between the distance and D and DS was 0.91 and 0.87, respectively—Fig. 3b).

### Scaling corrects adaptive sampling when estimating trial difficulty

**Fig. 4:**
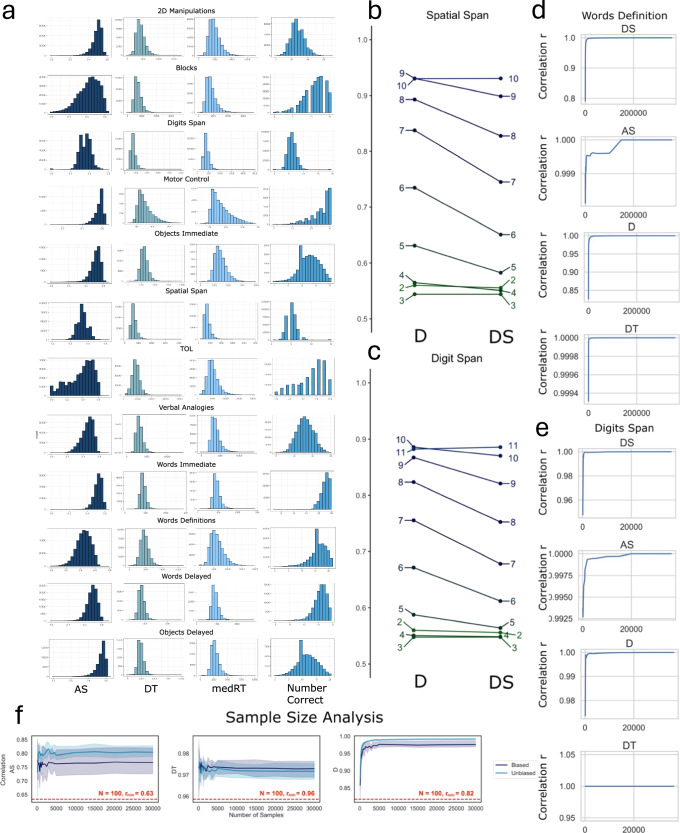
IDoCT validation through cross-participants distributions, demographic’s associations and sample size analysis. **a** Distributions of the estimated measures of AS and DT, as well as of the raw summary scores RT and Number Correct (or accuracy), across all tasks. AS is characterised by a distribution with negative skew, whereas DT by a distribution with a positive askew. The distribution of AS resembles Number Correct, while the distribution of DT resembles median RT. **b** Trials with higher number of squares were affected by the scaling due to the original estimated difficulty scores D being exclusively based on the performance of participants with high ability. **c** Trials with higher number of digits were affected by the scaling due to the original estimated difficulty scores D being exclusively based on the performance of participants with high ability. **d** Correlation between the measures of AS, DT, D and DS estimated using the full sample in Words Definitions and using subsamples of different sizes. **e** Correlation between the measures of AS, DT, D and DS estimated using the full sample in Digits Span and using subsamples of different sizes. **f** Results of sample size analysis using simulated data. The model can successfully predict the measures of AS, DT and D with as little as 100 samples and with a drop of performance of only respectively 12%, 2% and 12%.

The predicted difficulty scales D of the tasks based on pseudo-random balanced sampling of trials were not significantly affected by the scaling (e.g., TOL, the Object and Words Memory tasks, Words Definitions, Verbal Analogies, 2DManipulations, Blocks and Motor Control), as shown in Table 2. On the other hand, tasks such as Digits Span and Spatial Span, which are characterised by a biased sampling of trials, resulted in different DS compared to D. In these tasks, the trial’s assignment is based on an algorithm characterised by a staircase approach, meaning that the difficulty of the following trial depends on the performance of the participant in the previous one. As a result, the most difficult trials are exclusively presented to the best performing participants, which results in the model capturing a data-driven measure of D for specific trials that is exclusive dependent on participants with high ability. The scaling addressed this issue by correcting D for the AS of the participants that were presented a specific trial. An example of the effect of scaling on Spatial and Digits Span is provided in Fig. 4b, c. As can be observed, after the scaling, completing trials with 11 digits was harder than completing trials with 10 in case of Digits Span. Similarly, in case of Spatial Span, completing trials with 10 squares to memorise was harder compared to trials with 9.

### AS and DT have expected distributions mostly centred around 0.5

The model was separately applied to the 12 tasks and consistently converged in 10 iterations when estimating the measure of AT, which was used to estimate the measure of cognitive ability AS and visuomotor latency DT. The mean AS and DT per task are reported in Table 2, and their distributions, together with the distributions of the raw summary scores (i.e., RT and Number Correct), are shown in Fig. 4a (correlation between AS, DT and raw summary scores is available in Supplementary Table 1). The model-derived estimates of DT were generally characterised by a gamma distribution with a positive skew, which reflects that a minority of participants had delayed raw response times. Conversely, AS was generally centred around 0.5 and was characterised by a gamma distribution with a negative skew, which is more similar to the raw accuracy scores. Notably though, the AS distributions were generally smoother than raw accuracy scores, and in the case of Motor Control and Words Memory Immediate, overcame the ceiling effects evident in the raw scores.

### The model can perform well with as little as 100 participants

The simulation analysis (Fig. 4f and Supplementary Figure 1) suggested that IDoCT can reliably capture measures of D, DS, AS and DT also when as little as 100 samples are used. Being able to obtain precise measures with a smaller sample size is fundamental, since large scale data collection is not always feasible. To confirm these results, IDoCT was applied to the Words Definitions and Digits Span tasks using a range of N samples between 100 and the maximum number of participants available (e.g., 36,392 for Digits Span and 358,894 for Words Definitions). Digits Span was selected as it is characterised by an adaptive staircase algorithm, with the most difficult samples being presented only to the best participants. On the other hand, Words Definitions consists of a relatively complex trial’s space characterised by 150 different types of trials (i.e., words). The correlation between the measures of D, DS, AS and DT obtained when using different subsamples and the full dataset are presented in Fig. 4d, e. As can be observed, when training IDoCT on as little as 100 samples, in case of Digits Span, the Pearson’s correlation between the IDoCT derived metrics using the subsample and the full sample was very high at 0.948, 0.974, 0.992, and 0.999 for D, DS, AS and DT respectively. Similarly, in case of Words Definitions, the minimum correlation obtained between the IDoCT derived metrics using the subsample and the full sample was 0.80, 0.826, 0.998, 0.999 for respectively D, DS, AS and DT. When repeating the subsample analysis (30 times) with different subsets of 100 participants, the results were consistent, and just a minimal drop in performance was observed. Specifically, the derived correlation was 0.99 ± 0.003, 0.999 ± 0.007, 0.74 ± 0.06 and 0.68 ± 0.08 for respectively DT, AS, D and DS in Words Definitions, and 0.86 ± 0.22, 0.72 ± 0.20, 0.97 ± 0.03, and 0.98 ± 0.03 for DT, AS, D and DS respectively in Digits Span.

These results suggest that IDoCT can obtain reliable measures of AS, DT, D and DS with as little as 100 samples, and that the minimum observations required for good performance is mainly defined by the scale of the trial space, with more trial types requiring more samples to estimate difficulty reliably.

### Older participants have higher DT and lower AS in all tasks except for the language task, where knowledge of words increases with age

The association between the extracted measures of AS and DT and different demographics variables were assessed to confirm their validity as, respectively, measures of cognitive ability and visuomotor latency. Specifically, AS should have substantial and interpretable associations with age, education and language. By contrast, DT should have significant associations with age alone. To achieve this, multiple linear regression models were implemented, where age in decades (reference category: age between 20–30), education (reference category: preGCSE) and language (reference category: other) were used as regressors, and AS and DT as outcomes to be predicted. The significance of each category was assessed using Analysis of Variance (ANOVA). For comparison, the same analysis was repeated for median RT and Number Correct (or accuracy), and the results are presented in Supplementary Tables 2 and 3.

**Fig. 5:**
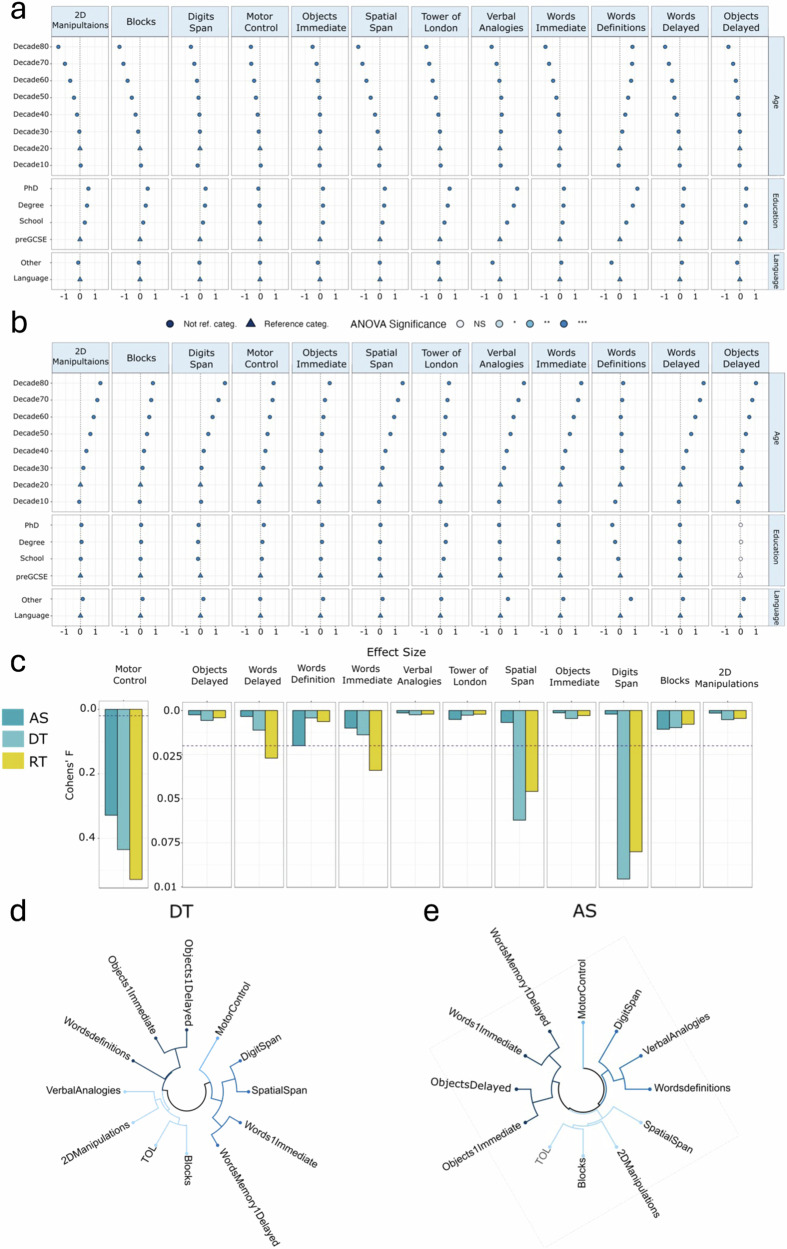
Demographics associations of age, education and language with AS. **a** The effect sizes in SD units of the different categories in age, education, and language are reported for all cognitive tasks when predicting AS. The colour represents whether the entire category was significant according to the ANOVA results, with each colour corresponding to different asterisks, where *p < 0.05, **p < 0.01, and ***p < 0.001. Triangles indicate reference categories. **b** The effect sizes in SD units of the different categories in age, education, and language are reported for all cognitive tasks when predicting DT. The colour represents whether the entire category was significant according to the ANOVA results, with each colour corresponding to different asterisks, where *p < 0.05, **p < 0.01, and ***p < 0.001. Triangles indicate the reference category. **c** The effect size of device was measured in terms of the Cohen’s F2. A score below 0.02 was considered as negligible. For all tasks, the effect of device was negligible in case of AS. For RT, it was in the small to medium range for Digits Span, Spatial Span and the Words Memory tasks. **d** Clusters obtained from applying hierarchical clustering to the pairwise correlation matrix of AS. **e** Clusters obtained from applying hierarchical clustering to the pairwise correlation matrix of DT.

The regression models were significant when predicting AS and DT derived from all cognitive tasks included in the study. Details on the results of each model are provided in Fig. 5a for AS and Fig. 5b for DT, where the effect sizes in standard deviation (SD) units of the different factors in each demographics category are reported.

Age, Education and Language had a significant association (*p* value < 0.001) with AS across all tasks (Fig. 5a). Specifically, an increase in age was associated with a decrease in AS for all tasks except Words Definition, where the opposite relationship was observed, which is as expected, because people acquire words throughout the lifespan^27^. The effect size of age spanned from medium to very large dependent on the task. At age 80, a change in SD units compared to the reference category (20s decade) of respectively −1.46 for 2DManipulations, −1.38 for Blocks, −0.59 for Digits Span, −0.61 for Motor control, −0.50 for Objects Immediate, −0.77 for Objects memory Delayed, −1.45 for Spatial Span, −0.91 for TOL, −0.55 for Verbal Analogies, 0.85 for Words Definitions, −0.97 for Words Immediate, and −0.99 for Words Delayed was observed.

As expected, the general trend was for participants with higher education levels to achieve higher AS scores. However, despite the effect being significant in all tasks, in Motor Control the effect size was small, with an absolute value that was overall smaller or equal to 0.1 SD units (0.05 SD). This was expected considered that Motor Control is primarily a motor task. On the other hand, both for Words Definitions and Verbal Analogies, having a PhD appeared to have a large association with AS, which was respectively 1.18 and 1.17 higher compared to participants with only GCSE as prior education. In case of the other tasks, having a PhD was associated to an increase in SD units for AS of 0.65 for TOL, 0.56 for 2DManipulations, 0.52 for Blocks, 0.43 for Objects Delayed, 0.40 for Digits Span, 0.34 for Spatial Span, and 0.27 for Words Immediate and Delayed.

Finally, being a non-native English speaker was a significant predictor of AS. However, its effect was in the small and negligible range for most of the tasks, namely Motor Control (0.002 SD), Blocks (−0.08 SD), 2D Manipulations (−0.12 SD), Digits Span (−0.09 SD), Objects Memory Immediate (−0.14 SD) and Delayed (−0.18), Words Memory Immediate (0.09 SD) and Delayed (0.14 SD), Spatial Span (−0.04 SD) and TOL (−0.11 SD). This is expected considered that these tasks mainly require spatial, motor and memory functions. On the other hand, in case of the two language tasks (i.e., Words Definitions and Verbal Analogies), participants with a mother tongue different from English were associated to respectively a 0.56 SD and 0.49 SD decrease in AS compared to English speaking participants.

When predicting DT, Age, Education and Language were statistically significant predictors in all tasks, except for Education in case of the Objects Memory Delayed (Fig. 5b). However, the effect sizes of language and education were predominantly in the small to negligible range. More specifically, an increase in age was associated to an increase in DT, and the effect size was always in the medium to large range, except for Words Definitions, where the oldest age bracket was associated with a small (0.19 SD) increase in DT. By comparison, participants at age 80 were associated to an increase in DT of 1.63 SD for Digits Span, 1.58 for Verbal Analogies, 1.57 and 1.41 for Words Memory Delayed and Immediate, 1.47 for spatial span, 1.34 for 2D Manipulations, 1.05 and 0.62 for Objects Memory Delayed and Immediate, 0.89 for Motor Control, 0.84 for Blocks, and 0.66 for TOL.

For education, the effect size was in the negligible to small range (<0.2 SD units) for all tasks except for Words Definitions and TOL, where having a PhD was associated to respectively a decrease of 0.54 SD and increase of 0.35 SD units in DT compared to participants without prior education. Finally, although language was a significant predictor of DT, the absolute effect size was below 0.2 for all tasks except Words Definitions (0.70) and Verbal Analogies (0.51).

### Device has a lower effect on the calculated measures of AS compared to the raw RT scores

We tested the effect of device on IDoCT estimated measures by training multiple linear regression models were the device label, together with other demographics confounders, were used as regressors and AS and DT as outcomes to be predicted. The analysis was repeated using the raw RT score, which tends to be the most sensitive to testing device, as the predicted variable for comparison (Fig. 5c). The device effect was measured in terms of the Cohens’ F2 scores calculated from the fitted multiple liner regression models. The effect of device on AS was smaller than for RT in almost all cases and was negligible (<0.02) in all tasks except Motor Control, which is specifically designed to vary motor demand across trials. By comparison, the effect of device for median RT for five tasks (i.e., Digits Span, Spatial Span, Motor Control, Words Immediate and Words Delayed) was in the small-medium range (>0.02). For DT, the device association was in the negligible range (<0.02) for all tasks except Digits Span, Spatial Span and Motor Control.

### The latent variable structure of AS is interpretable

Performance on tasks that involve similar combinations of cognitive processes are expected to correlate more highly than tasks that involve different cognitive processes. Relatedly, tasks with a similar visual, motor and confounding cognitive components are expected to have more correlated DT measures. To validate this, hierarchical clustering, which does not require a pre-defined number of clusters, was applied on Pearson’s correlation matrices of AS and DT (Fig. 5d, e). According to the results of AS, 4 major groups could be identified, with clearly interpretable similarities. Specifically, one cluster comprised the four measures of memory performance, these being Immediate and Delayed Object and the Words recognition. These further clustered by stimulus type (objects vs words) as opposed to recognition timeframe (immediate vs delayed). Another cluster included all language problem solving tasks, e.g., Words Definitions, Verbal Analogies and Digits Span (which involves the phonological loop^28^). The next cluster included all cognitive tasks that measure different aspects of spatial cognition, i.e., Spatial Span, 2D Manipulations, TOL and Blocks. Finally, Motor Control was separated as distinct from all others. Therefore, AS measures clustered according to the type of cognitive demand that the tasks were designed to measure.

For DT (Fig. 5d), 4 major clusters were identified, but the similarity cognitively was less clear, which is unsurprising as device latency and motor response complexity have more of a role. Specifically, Spatial Span and Digits Span and the Word-Memory tasks formed pairs within one cluster. The two spatial planning tasks and the Blocks and Verbal Analogies tasks formed two pairs within a second cluster. Words Definitions and Objects Memory Immediate and Delayed Recognition formed a third cluster. Finally, Motor Control was separated into one task cluster.

### Factor analysis on AS identifies more specific and meaningful cognitive domains compared to raw summary scores

**Fig. 6:**
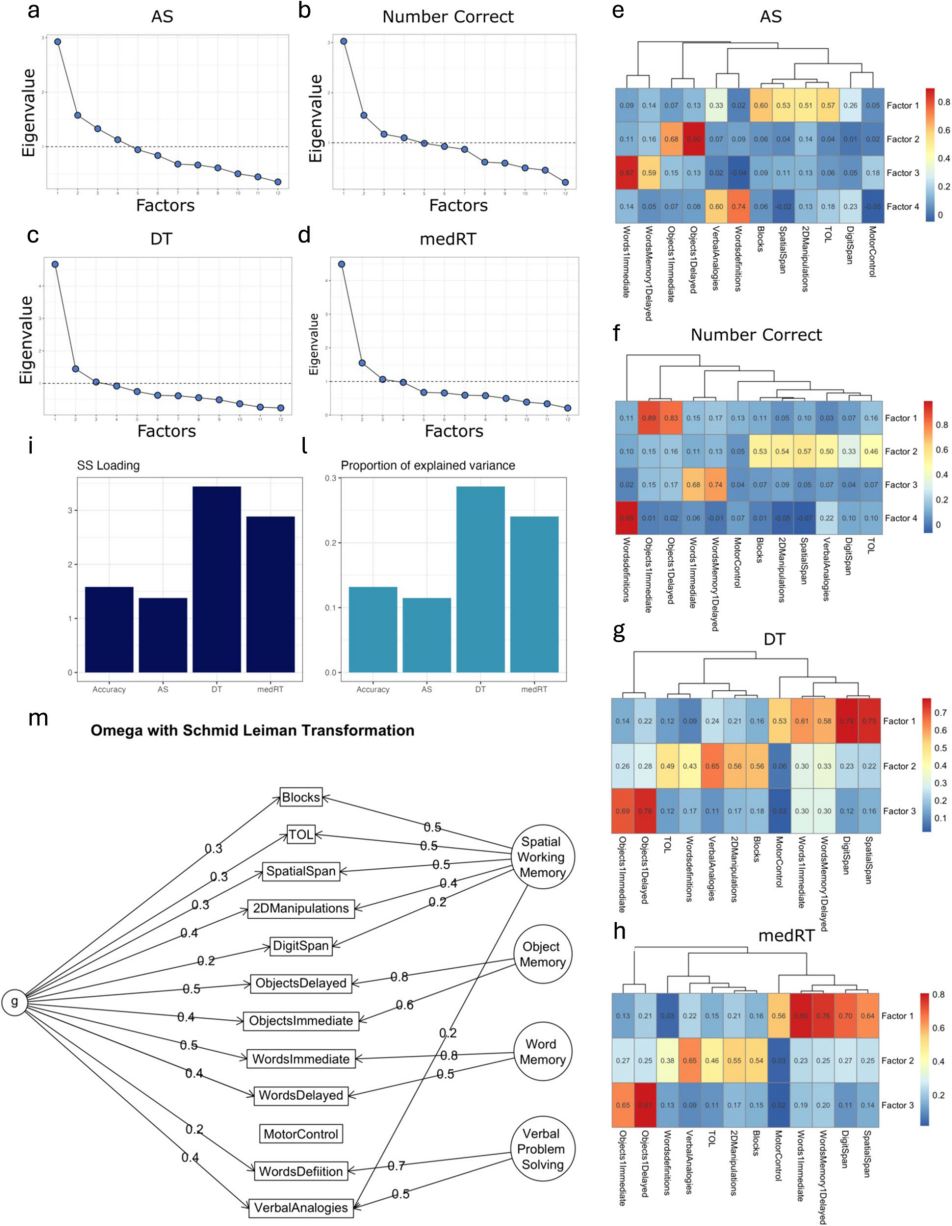
Psychometric and g-factor analysis. Scree plots (Factors vs Eigenvalue) for AS (**a**), Number Correct (**b**), DT (**c**) and RT (**d**). The dotted line corresponds to an Eigenvalue equal to 1. **e** AS - factor analysis with varimax rotation. **f** Number Correct—factor analysis with varimax rotation. **g** RT—factor analysis with varimax rotation. **h** DT—factor analysis with varimax rotation. **i** The sum of squares loadings from the g-factor analysis were 1.35, 2.88, 3.44 and 1.58 for AS, DT, RT and accuracy respectively. **l** Percentage of variance explained by the g-factor for AS, DT, RT and accuracy were respectively 11%, 24%, 28%, 13%. The measure of AS had the least amount of variance explained by a 2nd order g-factor. **m** Schmid–Leiman factor model for AS.

A factor analysis with varimax rotation was computed separately on AS, DT and the raw measures Number Correct (or accuracy) and RT to evaluate whether tasks that are supposed to capture similar cognitive domains load on the same factor. The number of factors per summary measure was selected according to the Kaiser criterion (eigenvalues > 1) (Fig. 6a–d), resulting in 3 factors for DT and RT and 4 factors for Number Correct and AS.

Four factors were identified with interpretable cognitive functions: specifically, F1) Spatial Planning and Working Memory (TOL, 2D Manipulations, Spatial Span, and Blocks), F2) Object Memory (Objects Immediate and Delayed Recognition), F3) Word Memory (Words Immediate and Delayed Recognition), and F4) Verbal Problem Solving (Verbal Analogies and Words Definitions). Motor Control did not have a high loading on any of the factor, which is expected given that it is the only task to measure the spatial accuracy of motor responses. On the other hand, in case of the raw summary measure Number Correct, the Verbal Analogy and Words Definitions tasks did not load on the same factor, with Verbal Analogies loading on the factor related to Working memory (Fig. 6e, f).

For DT, three factors were identified (Fig. 6g, h). The tasks with the highest loading on the first factor were the Digits and Spatial Span, Words Memory Delayed and Immediate, and Motor Control. The second factor was mainly affected by the DT scores in all the remaining language (i.e., Words Definitions and Verbal Analogy) and spatial related tasks (i.e., Blocks, TOL, and 2Dmanipuation). The two Objects Memory tasks had the highest loading on the third factor. The factors identified on RT were comparable to DT.

### The g factor explains only 11% of the variance in AS

To assess the scale of the ‘g’ factor in the raw and IDoCT measures, we applied the Schmid-Leiman transformation to the accuracy, RT, AS and DT correlation matrices. The proportion of variance explained by the g factor for AS, DT and accuracy and RT, was respectively 11%, 28%, 13%, 24% (Fig. 6i, l). Therefore, as intended, AS provided the most discrete fractionation of cognitive abilities (Fig. 6m).

## Discussion

Our results demonstrate that by applying data-driven modelling to detailed trial-by-trial recordings from carefully designed computerised tasks, IDoCT can generate detailed cognitive profiles encompassing sensitive and distinct measures of planning, reasoning, attention, and memory capabilities from assessments that are conducted online under unsupervised conditions. This method has a broad range of potential applications in both research and clinical contexts.

To date, the substantial design and validation efforts that have gone into developing online cognitive assessment software have not been accompanied by the modelling work that is necessary to maximise the utility of the resultant data. This is important because, although online data has the advantage of scalability, repeatability and cost-efficiency, there are particular challenges that make these data not trivial to use. Specifically, these challenges arise from the differences in the interfaces and screens of the variety of devices that people are tested on. These challenges add to those that are more generic to cognitive assessment, e.g., issues in accounting for basic visuomotor speed, calibrating task difficulty scales, accounting for confounding cognitive abilities and handling speed accuracy trade-offs when seeking to estimate targeted cognitive abilities. Improving cognitive ability estimates in terms of accuracy and cognitive process specificity has the potential to produce stronger and more interpretable correlations in association studies of registers, cohorts and biobanks, where large population scale is required. It can also aid clinical trials^29^ and clinical practice, where the precision of individual patient assessment profiles is critical^30^.

The current study provides converging evidence that IDoCT can help achieve this potential. Regarding distributions, it improves those obtained via raw summary scores by combining response time and accuracy measures, thereby handling speed accuracy trade-offs, overcoming ceiling effects, and outputting smoother and more precise popular distributions (e.g., positively and negatively skewed gamma distributions for AS and DT respectively) relative to summary accuracy scores. In the process, it re-estimates the difficulty scales that relate trial-by-trial variability to cognitive dimensions, enabling them to be interpreted in a purely data-driven manner and reducing bias when estimating abilities from performance.

Most critically, IDoCT successfully accounts for device, visuomotor and confounding cognitive components of performance, as demonstrated by the negligible scaled association of the cognitive score estimates with differences in testing device alongside strong and interpretable associations with demographic factors. The notable exception was Motor Control; however, this is unsurprising because the main requirement for tasks to be suitable for IDoCT is that cognitive dimensions and perceptual or motor demands are manipulated in a dissociable manner across trials. Indeed, this requirement is a pillar of good task design in experimental psychology methodology. The Motor Control task is designed to use distance between consecutive targets on the screen as the manipulated difficulty variable; consequently, it is sensitive to the absolute size of the stimuli on the screen, a variable that cannot be controlled through the web browser.

When these conditions are met, our results confirm that IDoCT produces stable trial difficulty and cognitive ability estimates across a variety of cognitive tasks with as few as 100 participants. Therefore, it can be applied in studies where data collection is not at the same unusual large scale as is available in our citizen-science database. Conversely, the limited computational resources required to train IDoCT on hundreds of thousands of participants makes it feasible for such large-scale studies, which we expect will become more common in the future as more research migrates to online deployment.

Regarding precision, a long-standing objective with relevance across psychometrics, intelligence testing and cognitive assessment has been to derive measures of cognitive abilities that are dissociable, that is, to produce detailed multivariate cognitive profiles as opposed to scores on a single monolithic scale^31,32^. Achieving this objective is complicated by the fact that cognitive tasks measure mixtures of abilities; therefore, they tend to produce measures that are correlated across population samples^33^. When performing a factor analysis across entire assessment batteries, these correlations form the basis of a general factor, which in intelligence testing is referred to as IQ or ‘g’.

For widely used intelligence tests like the Wechsler Adult Intelligence Scale (WAIS), the Cattell Culture Fair Intelligence Test, or the Raven’s Progressive Matrices, the ‘g’ factor has been found to account for a substantial proportion of the total variance in performance. Typically, the ‘g’ factor explains in the order of 40% to 50% of the variance in scores on these tests^34,35^. We can expect performance variability that relates to device, basic visuomotor processing and confounding cognitive processes to correlate across tasks, thereby further inflating the ‘g’ factor. Accordingly, when applied to one of the few online batteries applied at similar scale to ours, exploratory factor analysis has even been shown to produce just a single ‘g’ factor^36,37^. This presents a problem for understanding the basis of observed impairments at the level of cognitive processes, and their differential associations, for example, with psychosocial factors or underlying brain systems.

To enable better measurement precision, the summary measures of our cognitive task battery focus on accuracy, not speed, as it is less sensitive to device. The former measures are also deliberately decorrelated at the design and validation stages to minimise the scale of the general intelligence factor^25^. This is evident in the factor analysis of raw scores, where the scale of the ‘g’ factor is favourable for accuracy but less so for response time. Notably, IDoCT successfully combined the accuracy and response time scores into a single, more precise, summary ability AS while reducing the ‘g’ factor relative to raw accuracy to explaining just 11% of the variance, as defined using a Schmidt-Liemen model^38^, which is to be expected since AS is supposed to capture task-specific, more granular, cognitive processes. Although AS appears to have only a 2% improvement in variance explained by the g-factor compared to accuracy, it is important to highlight that AS actually combines both RT and accuracy into one unique measure, and the variance explained by the g-factor for reaction time is 13% higher. Furthermore, the resultant factor structure comprised four latent variables with, in our opinion, more interpretable loadings relative to the raw accuracy or response time scores. Specifically, the Verbal Analogies and Words Definitions tasks both loaded on the same factor, in contrast to the raw accuracy, suggesting that AS yields factors that capture more precise cognitive domains. Conversely, the DT estimates showed an increased ‘g’ factor relative to raw RT, which is to be expected as it is intended to capture the device-related performance variance and more general aspects of intelligence (e.g., cognitive processes that are not task specific).

Given the superior specificity of IDoCT estimates, a sensible future direction is to determine whether AS and DT correlate with cognitive and motor systems respectively in the context of large brain-wide imaging association studies, and whether AS measures of different tasks functionally fractionate cognitive brain networks^39,40^. Also, research should evaluate the performance of IDoCT on cognitive data from patients affected by complex multi-system conditions such as Multiple Sclerosis, Parkinson’s disease and stroke, to determine how well it can differentiate between visuomotor vs. cognitive impairments, and whether it can provide improved measures for clinical research and healthcare applications.

Future methodological directions include further developing the IDoCT approach to handle tasks that are designed to manipulate the difficulty of multiple distinct cognitive processes, and to dissociate visuomotor and device related components of performance from each other. The former may be achieved through modifications to the model to include how multiple cognitive scales relate to different latent abilities – an approach that could build on principle from multiple item response theory. The latter may require the development of online psychophysiological tasks which manipulate different aspects of visuomotor difficulty, that is, complementing the Motor Control task used in the current study, and applying hierarchal modelling.

In conclusion, IDoCT provides a simple, flexible and validated modelling approach that can improve the measurement accuracy and precision of automated online cognitive assessment scores from moderate to large scale studies in a computationally efficient and data driven manner, with broad potential applications.

## Methods

We propose an iterative method called IDoCT (Iterative Decomposition of Cognitive Tasks) to disentangle the motor, device and cognitive components of performance in online cognitive tasks. The model was tested and validated on simulated data and 12 different cognitive tasks implemented using the Cognitron software, and whose data were collected as part of the Great Britain Intelligent Test (GBIT)^24^.

### Great British intelligent test

GBIT is a large-scale population-based study whose aim is to collect information about the mental health, wellbeing, behaviour and cognition in the general population, with a specific focus to the UK^24^. It was first launched in December 2019, and by the time of the study it had completed 5 timepoints of data collection, which started respectively in December 2019, May 2020, December 2020, June 2021, and January 2022. Each data collection timepoint consists of online questionnaires, and cognitive tasks, that could be completed on devices people already own (e.g., mobile phones, desktop computers or tablets). Different sets of tasks are used at each timepoint of data collection, with a subset presented to all participants at all timepoints, while the remainder were assigned to different participants according to a random sample approach. The GBIT data were collected using the Cognitron software and tasks, which were validated in multiple research studies on healthy and clinical populations^3,7,9,10,41^.

The study was approved by the Imperial College Research Ethics Committee (17IC4009), complied with ethical regulations and participants provided informed consent prior to starting the study.

### Data

In this study, we analysed 12 cognitive tasks that assess different cognitive domains, namely semantic reasoning (i.e., Verbal Analogies), mental spatial manipulation (i.e., 2D Manipulations), immediate and delayed memory of words (i.e., Words Memory) and objects (i.e., Objects Memory), spatial planning (i.e., Tower of London), spatial short-term memory (i.e., Spatial Span), digit short-term memory (i.e., Digits Span), reaction time (i.e., Motor Control), language (i.e., Words Definitions), and executive function (i.e., Blocks). A brief description of the different tasks is provided in Fig. 2. The data for the cognitive tasks consisted of trial-by-trial measures of RT, and accuracy, and of trial-by-trial descriptions of the task design. In addition, to validate the results of the model, we used demographics data (i.e., language, sex, ethnicity, occupation, handedness, education, and residence), and information about the device used while completing the online cognitive assessment.

### Pre-processing of demographics and questionnaire data

The demographics data underwent an initial stage of pre-processing, during which inconsistencies across timepoints were detected and addressed, and the different features were grouped into meaningful categories. In this study, only participants that completed one timepoint (across the five available) were included, which means that inconsistencies in demographics across timepoints did not affect the results of the analysis.

The language of participants was grouped into “English” and “Other”. The education was categorised into four major groups, namely pre-GCSE, School, Degree and PhD. The ethnicity was defined according to the commonly used categories in the Census, which are (1) Asian or Asian British, (2) Black, Black British, Caribbean or African, (3) Mixed or multiple ethnic groups, (4) White, and (5) Other ethnic groups. Residence was grouped into “United Kingdom” and “Others”. Device labels were grouped into 8 categories: Apple computer, Windows computer, Linux computer, Apple phone, Apple tablet, Android phone, Android tablet, and Chrome computer. Age values below 16 and above 90 were marked as missing, since likely to be recording errors, and age was binned into decades (from decade 10 to 80, where decade 10 included all participants with age between 16 and 20). Occupation was grouped into ‘Worker’, ‘Retired’, ‘Student’, ‘Unemployed/Looking for work’, ‘Homemaker’, ‘Disabled/Not applicable/Sheltered employment’, ‘Unknown’.

A measure of RT was collected for each demographics’ question, which represents the time required by participants to provide an answer. A lower tail threshold was applied on the distribution of the RT across all participants for each demographics question. The aim was to detect answers where the RT was so low to suggest that participants did not take the time to properly read the questions before selecting an answer. The thresholds for the lower tail of the demographics RT distributions were detected using a conservative automated approach that is based on the height of the histograms of the distribution plot. No filtering was applied on the upper tail of the RT distributions. This filtering was computed independently for each timepoint. More information on the automated algorithm and exact thresholds applied to the demographics features is available in Supplementary Table 4.

### Processing of cognitive data

The outliers in the trial-by-trial RTs distributions were removed. Specifically, the across samples distributions of RT values were filtered in order to exclude the answers that were too fast or too slow, as these were a sign of either cheating or not properly engaging with the cognitive assessment. These thresholds were identified by manually checking the RT distributions of each cognitive task. The exact thresholds, together with the percentage of trials excluded as a result of the filtering, are reported in Supplementary Tables 5 and 6. If participants completed the same task across multiple timepoints, they were dropped to prevent detecting a learning curve. The number of participants excluded per task because of this filtering is reported in Supplementary Table 6.

In case of Spatial and Digits spans, all trials with a target length longer than 10 and 11 respectively were removed, as it was a sign of cheating (e.g., participants writing down the numbers or locations of the squares).

### Measure of trial difficulty D

The IDoCT model takes as input the trial-by-trial responses of participants, and specifically their reaction time RT (e.g., how long it took them to answer each trial), their accuracy (e.g., whether the response in each trial was correct, or a measure of how correct each response was) and a definition of trial difficulty D, which is task specific. In order to obtain a data-driven measure of D, a fixed-point iteration must be computed.

In the first iteration, given the three inputs, the performance of participant *i* for trial *t*, *P*(*i*, *t*), can be measured in terms of a binary or continuous accuracy, depending on the task. Binary accuracy occurs when participants can either respond correctly or not to a specific trial. On the other hand, continuous accuracy specifies to what extent the response was correct.

In the case of binary accuracy, *P(i, t)* can be defined as described in Eq. (1):

1 $$P\left(i,\,t\right)=\left\{\begin{array}{ll}\qquad\qquad0, \, {if\; wrong\; answer}\\ 1-\frac{{RT}\left(i,t\right)}{{{RT}}_{\max }}, \, {if\; right\; answer}\end{array}\right.$$

where RT(*i*, *t*) is the reaction time of participant *i* in trial *t*, *RT*_max_ is the maximum RT across all trials and all participants. The performance is set equal to 0 in case of the wrong answer because the RT is not representative of people ability in a given trial. Indeed, when they don’t know the answer, people can decide to spend longer times thinking about a trial, or reply immediately to move to the next one.

In case of continuous accuracy, instead, *P*(*i*, *t*) can be measured as in Eq. (2):

2 $$P\left(i,{t}\right)=\left(1-\frac{{RT}\left(i,{t}\right)}{{{RT}}_{\max }}\right)* Acc\left(i,{t}\right)$$

where *Acc*(*i*,*t*) is a continuous accuracy measure (between 0 and 1) of participant *i* in trial *t*. The *RT*_max_ is simply used as a scaling factor, which means that it shouldn’t impact significantly the results. Even if *RT*_max_ was an outlier, the ranking of the participants’ abilities would remain the same. However, it is appropriate to check for outliers before applying the model.

Once *P*(*i*, *t*) is defined, the difficulty of a trial *D*(*t*) can be calculated as shown in Eq. (3):

3 $$D\left(t\right)=\,\frac{1}{\mathop{\sum }\nolimits_{i=1}^{N}T(i,\,t)}\mathop{\sum }\limits_{i=1}^{N}\mathop{\sum }\limits_{j=1}^{T\left(i,\,t\right)}1-P\left(i,\,t,j\right)$$

where *T*(*i*, *t*) is the number of times participant *i* completed the same trial *t*, which accounts for cases in which the same trial was presented multiple times to the same participant *i*. *N* is the total number of participants that were presented trial *t.* *j* is a counter that allows to sum all the performances for participant *i* in a given trial *t*. The first iteration of the model assumes that *D*(*t*) depends on the performance *P*(*i*, *t*) of participants across different trials, but not vice versa. More specifically, it assumes that the difficulty of each trial is always equivalent to 1, when measuring *P*(*i*, *t*).

From the second iteration, a mutual recursive definition is created, in which *D*(*t*) and *P*(*i*, *t*) both depend on each other, and the definition of *P*(*i*, *t*) is updated as shown in Eqs. (4) and (5).

4 $$P\left(i,\,t,\,n\right)=\left\{\begin{array}{ll}\qquad\qquad\qquad\qquad\quad0, \, {if\; wrong\; answer}\\ \left(1-\frac{{RT}\left(i,t\right)}{{{RT}}_{\max }}\right)* D(t,\,n), \, {if\; right\; answer}\end{array}\right.$$

5 $$P\left(i,{t},{n}\right)=\left(1-\frac{{RT}\left(i,{t}\right)}{{{RT}}_{\max }}\right)* Acc\left(i,{t}\right)* D(t,\,n)$$

Respectively for binary and continuous accuracy. *D*(*t*, *n*) where n = 0 corresponds to the first iteration. In this case, *D*(*t*, 0) = 1 for all trials *t*, which means, as previously mentioned, that all trials *t* are assumed to have equivalent and maximum difficulty *D*. The final measure of *D*(*t*) is obtained when *D*(*t*) and *P*(*i*, *t*) converge, as shown in Eqs. (6) and (7).

6 $$D\left(t\right)=D\left(t,\,n\right)\,{with}\,n\,{such\; that}\,D\left(t,n+1\right)=D(t,\,n)$$

7 $$P\left(i,\,t\right)=P\left(i,\,t,n\right)\,{with}\,n\,{such\; that}\,P\left(i,\,t,n+1\right)=P\left(i,\,t,n\right)$$

### Measure of ability A

To obtain a measure of *A*(*i*) (e.g., ability) for each participant *i*, *RT*(i, t) must be described in terms of two separate components, namely the answer time *AT* for participant *i* at trial *t*, *AT*(*i*,*t*), and the delay time *DT* for participant *i*, *DT*(*i*) (Eq. (8) and (9)).

8 $${RT}\left(i,\,t\right)={AT}\left(i,\,t\right)+{DT}(i)$$

With the constraint

9 $$0 \,<\, {DT}\left(i\right)\le {{DT}}_{\max }(i)$$

where *DT*_max_*(i)* is defined as *RT*_min_*(i)*, which is the smallest *RT* across all trials for each participant *i* (Eq. (10))

10 $${{DT}}_{\max }\left(i\right)={RT}\left(i,\,t\right)\,{for\; the}\,t\,{such\; that\; RT}\left(i,\,t\right)\,{is\; the\; smallest}$$

Both *DT*(*i*) and *AT*(*i*, *t*) are measured through an iterative approach. According to the new definition of *RT*(*i*, *t*), the measure of *P*(*i*, *t*) becomes as follows (Eqs. (11) and (12)):

11 $$P\left(i,\,t\right)=\left\{\begin{array}{ll}\qquad\qquad\qquad\qquad\qquad0, \, {if\; wrong\; answer}\\ \left(1-\frac{{AT}\left(t,i\right)+{DT}\left(t,\,i\right)}{{{RT}}_{\max }}\right)D(t), \, {if\; right\; answer}\end{array}\right.$$

For binary accuracy, and

12 $$P\left(i,{t}\right)=\left(1-\frac{{AT}\left(t,i\right)+{DT}\left(t,\,i\right)}{{{RT}}_{\max }}\right)* Acc\left(i,{t}\right)* D(t)$$

For continuous accuracy.

The answer time *AT*(*t, i*) can be calculated as shown in Eqs. (13) and (14).

13 $${ATN}\left(i,\,t\right)=\left(1-A\left(i\right)\right)* D(t)$$

14 $${AT}\left(i,\,t\right)={ATN}(i,\,t)* ({RT}(i,\,t)-{{DT}}_{\max }\left(i\right))$$

where *A*(*i*) corresponds to the ability of participant *i*, *ATN*(*i*, *t*) is the measure of *AT*(*i*, *t*) normalised between 0 and 1, and *D*(*t*) is the previously established measure of trial difficulty. In the first iteration of the model, the measure of *AT*(*i*, *t*, *n*), where the iteration number *n* is equal to 0, is initialised as *AT*_min_*(i)*. Instead, *DT*(*i, 0*) is initialised as *DT*_max_(*i*). *AT*_min_(*i*) can be calculated as shown in Eq. (15).

15 $${{AT}}_{{min}}(i,t)={RT}(i,\,t)-\,{{DT}}_{max }(i)$$

The first iteration assumes that *A*(*i*) is maximum for all participants *i* and is, therefore, always equivalent to 1. From the second iteration, a mutual recursive definition of *A*(*i*) and *P*(*i*, *t*) is introduced by defining *A*(*i*) as (Eqs. (16), (17), and (18)):

16 $$B\left(i,\,t\right)=B\left(i,\,t-1\right)+P(i,t)$$

17 $${BN}\left(i,N\right)=\frac{\mathop{\sum }\nolimits_{t=1}^{N}B\left(i,t\right)}{N}$$

where *B*(*i*, 0) = 0 and *BN*(*i*, *N*) correspond to the cumulative performance *P* up to trial *N*. Once the cumulative performance is calculated, *A*(*i*) can be measured as its overall average:

18 $$A\left(i\right)={BN}(i,Q)$$

where *Q* corresponds to the total number of trials presented to participant *i*. From the second iteration, *A*(*i*) depends on *P*(*i*, *t*), which depends on *AT*(*i*, *t*) that, in turn, depends on *A*(*i*), creating a mutual recursive definition. The definition of *P*(*i*,*t*) can be updated as follows in case of binary accuracy (Eq. (19)):

19 $$P\left(i,\,t\right)=\left\{\begin{array}{ll}\qquad\qquad\qquad\qquad\qquad\qquad\qquad\qquad\qquad\qquad0, \, {if\; wrong\; answer}\\ \left(1-\,\frac{\left(1-A\left(i\right)\right)* \,D\left(t\right)* \left({RT}\left(i,\,t\right)-{{DT}}_{\max }\left(i\right)\right)}{{{RT}}_{\max }}-\frac{{DT}\left(i\right)}{{{RT}}_{\max }}\right)D(t), \, {if\; right\; answer}\end{array}\right.$$

And as follows in case of continuous accuracy (Eq. (20)):

20 $$P\left(i,{t}\right)=\left(1-\frac{\left(1-A\left(i\right)\right)* \,D\left(t\right)* \left({RT}\left(i,\,t\right)-{{DT}}_{\max }\left(i\right)\right)}{{{RT}}_{\max }}-\frac{{DT}\left(i\right)}{{{RT}}_{\max }}\right)* Acc\left(i,{t}\right)* D(t)$$

Since there is a recursive definition between *P* and *A*, a fixed-point computation is required. The final *P* and *A* converge as shown in Eqs. (21) and (22).

21 $$P(i,t)=P\left(i,t,\,n\right)\,{with}\,n\,{such\; that}\,P\left(i,t,n+1\right)=P(i,t,\,n)$$

22 $$A\left(i\right)=A\left(i,\,n\right)\,{with}\,n\,{such\; that}\,A\left(i,n+1\right)=A\left(i,n\right)$$

At each iteration, *DT*(*i,t*) is calculated using the Eqs. (23) and (24).

23 $${DT}\left(i,\,t\right)={RT}\left(i,\,t\right)-{AT}(i,\,t)$$

24 $${DT}\left(i\right)=\,\mathop{\sum }\limits_{t=1}^{Q}\frac{{DT}(i,\,t)}{Q}$$

where *Q* corresponds to the total number of trials presented to participant *i*.

### Measure of specific ability AS

Once *DT*(*i*) and *AT*(*i*, *t*) are calculated, a measure of specific performance *PA*(*i*, *t*) can be obtained, which corresponds to the performance based on the *RT* corrected for the delay time *DT*. *PA*(*i*, *t*) can be measured as shown in Eq. (25) in the case of binary accuracy.

25 $${PA}\left(i,\,t\right)=\left\{\begin{array}{ll}\qquad\qquad\qquad\qquad0, \, {if\; wrong\; answer}\\ \left(1-\frac{{AT}\left(i,t\right)}{{{AT}}_{\max }}\right)* D(t), \, {if\; right\; answer}\end{array}\right.$$

And with Eq. (26) in case of continuous accuracy:

26 $${PA}\left(i,t\right)=\left(1-\frac{{AT}\left(i,t\right)}{{{AT}}_{\max }}\right)* Acc\left(i,\,t\right)* D(t)$$

A measure of specific ability *AS*(*i*), which corresponds to the ability only dependent on *AT*(*i*, *t*) and therefore *PA*(*i*, *t*), can be calculated as shown in Eqs. (27) and (28).

27 $${BS}\left(i,\,t\right)={BS}\left(i,\,t-1\right)+{PA}(i,t)$$

28 $${BNS}\left(i,N\right)=\frac{\mathop{\sum }\nolimits_{t=1}^{N}{BS}\left(i,t\right)}{N}$$

where *BS*(*i*, 0) = 0 and *BNS*(*i, N*) corresponds to the cumulative performance *PA* up to trial *N*. Once the cumulative performance is calculated, *AS*(*i*) can be measured as its overall average (Eq. (29)).

29 $${AS}\left(i\right)={BNS}(i,Q)$$

where *Q* corresponds to the total number of trials presented to participant *i*.

### Scaled measure of difficulty DS

The model should be able to implicitly correct itself for the potential biased sampling that can occur if high difficulty words are presented exclusively to participants with higher specific ability. This is thanks to the step in which, to measure the answer time, the measure of difficulty is corrected according to the ability of participants. However, this will lead to appropriate measures of delay time and specific ability, but still potentially a biased scale of trial difficulty. In order to address this issue, the measure of difficulty can be corrected according to the specific ability of participants that completed the trials based on which it was calculated. A new measure of scaled difficulty, DS, can be calculated as shown in Eq. (30).

30 $${DS}\left(t\right)=\frac{\mathop{\sum }\nolimits_{i=1}^{N}{AS}\left(i\right)* D\left(t\right)}{N}$$

*DS*(*t*) is finally scaled according to the original *D*(*t*) range. *N* corresponds to the total number of participants that where presented trial *t*.

### IDoCT final estimates

As a result of the model, four major estimates are extracted: a measure of specific ability AS and delay time DT for each participant, and a difficulty D and scaled difficulty DS score for each type of trial. AS is supposed to represent a measure of cognitive ability which is specific to the cognitive domain assessed in the cognitive task, while DT is expected to capture any visuomotor and cognitive process that is not specific to the cognitive domain of the task, as well as device latency.

### IDoCT implementation

A binary accuracy measure was used for Blocks, Immediate/Delayed Words Memory, 2D Manipulations, Digits and Spatial Span, Words Definitions and Verbal Analogies. Instead, a continuous accuracy was applied in case of the Immediate and Delayed Object Memory tasks, and Motor Control. In case of Motor Control, the continuous accuracy was defined as the distance from the centre of the target and the location where participants pressed on the screen. On the other hand, for the Objects Memory tasks, the continuous accuracy was calculated according to the following criteria: a score of 3 was assigned if the correct item, with the right pose and orientation was selected, a score of 2 was given if the item and pose were correct but not the orientation, and a score of 1 was assigned if the item chosen was correct, but in the wrong pose and orientation. The orientation was irrelevant if the pose or item were wrong, and the pose was irrelevant if the item was incorrect. The continuous accuracy was always normalised between 0 and 1.

The definition of trial difficulty was task specific. It corresponded to the word presented at each trial in case of Words Definitions, the words that participants were supposed to remember in case of the Words Memory Tasks, the combination of the start and end rings configuration for TOL, the distance from the previous target for Motor Control, the number of digits or squares to remember in case of respectively Digits and Spatial Span, the semantic distance and analogy type in Verbal Analogies, the combination of the location of the answer and of its angle of rotation in 2D Manipulations, the combination of the pose, item and category of the objects that participants were required to recall in case of the Objects Memory tasks, and the combination of the number of moves and drops in Blocks.

The reaction time corresponded to the time required to complete a trial for all tasks except Digits and Spatial Span and Blocks. In Digits Span, Spatial Span and Blocks the reaction time was the average time to select a digit, square and block respectively.

### Sample size analysis using online data

IDoCT was applied to Digits Span and Words Definitions using different subsets of N samples (from 100 up to the maximum number of participants available per task), to validate how many samples are required in order for the model to reliably capture measures of cognitive ability and delay time. The Pearson’s correlation between the measures of AS and DT obtained using the subsets and the full dataset were calculated.

### Age, education, language and occupation association

A multiple linear regression model was fitted using as outcomes to be predicted the measure of AS and DT, and as regressors different hot encoded demographics variables (e.g., age in decade, education, and language). Information about ethnicity, sex, residence and handedness were included in the regression model as confounding factors. The reference categories were 20 years old for age, pre-GCSE for education, and English for language. For each categorical variable, the effect size was measured, which corresponds to the beta coefficient obtained from the regression model divided by the standard deviation of either AS or DT. Two separate regression models were fitted for each cognitive task respectively for AS and DT. In order to obtain a measure of significance of each category a type 2 ANOVA of the regression model was conducted. An alpha threshold of 0.01 was used to classify each category as significant.

### Association with device

A multiple linear regression model was fitted using as outcomes to be predicted the measures of AS, DT, and the raw summary score RT (calculated as the median RT across all trials), and as regressors different hot encoded demographics variables (e.g., age in decade, education, language, occupation, ethnicity, sex, residence and handedness), as well as device. The same reference categories used when studying the association of AS and DT with different demographics were applied. The ANOVA of the regression model was calculated, and a measure of Cohen’s F2 coefficient was obtained for the device category to assess the effect of the device on AS, DT and RT. A Cohen’s F2^42^ score smaller or equal to 0.02 was considered as negligible. To calculate the Cohen’s score Eq. (31) was used:

31 $${f}^{2}=\frac{1-{R}^{2}}{{R}^{2}}$$

Where the *R*^2^ corresponds to the coefficient of determination obtained from the multiple linear regression models.

### Measures of correlation and clustering across cognitive tasks

Some of the participants included in the study completed multiple cognitive tasks in one session, which resulted in having participants with AS and DT measures across tasks. These measures were used to compute a pairwise correlation matrix, in order to account for the sparsity of the data. Hierarchical clustering was applied on the obtained correlation matrix for AS and DT, using complete linkage as a method, to assess whether the tasks could be grouped into different clusters based on the measures of AS and DT.

### Factor analysis and g-factor

Factor analysis with a varimax rotation was applied to the pairwise correlation matrix of DT and AS obtained by correlating the scores of different participants across different cognitive tasks. The Kaiser Criterion was used to select the optimal number of factors for both DT and AS, which implies that only the factors with an eigenvalue bigger than 1 were kept for analysis. This resulted in 4 factors for AS, and 3 for DT. The Schmidt Leiman Transformation (SL), which is a commonly used tool for bifactor analysis^38^, was applied to the results of the factor analysis in order to identify a general factor (g-factor) measure^43^. The sum of squares loading of the g-factor was extracted. The same analysis was repeated for the raw measures of RT and accuracy to assess and compare the influence of the g-factor on the different measures of performance, and to evaluate the latent factors identified by the raw measures.

### Software

The analysis was conducted in Python (3.9.13) and R (4.0.1). The plots were generated either in Python using seaborn and matplotlib, or in R using ggplot2. The model’s code is available open source at https://github.com/valegiunchiglia/IDoCT.

## Supplementary information

Supplementary Material

## Data Availability

The GBIT data will be released in pseudonymised format at the point of publication. The code for the model will be released open access on GitHub at time of publication.

## Code availability

The code for the model will be released open access on GitHub at time of publication (https://github.com/valegiunchiglia/IDoCT).
