# Supplementary material for: An iterative approach for estimating domain-specific cognitive abilities from large scale online cognitive data
Source: NPJ Digit Med. 2024 Nov 19;7:328. doi: 10.1038/s41746-024-01327-x (PMC11576988; doi:10.1038/s41746-024-01327-x)
Supplement: Supplementary file 1 — Supplementary Material [file 41746_2024_1327_MOESM1_ESM.pdf]

Supplementary Material

1. Simulation analysis with random and adaptive schema

A simulation analysis was conducted to evaluate the prediction of the models compared to a simulated ground truth. As part of the simulation, 340 words and 30000 participants were generated, to resemble a cognitive task similar to Words Definitions. D(t) was simulated by sampling numbers from an exponential distribution with scale equal to 10, and by forcing to sample values in the range of 0 and 1. AS(i), instead, was obtained by sampling numbers from a gaussian distribution with scale equal to 0.05 and log equal to 0.5. Finally, simulated DT(i) measures were generated by sampling values from a negative bimodal distribution with p equal to 0.2 and n equal to 10. RT for each participant and each trial (e.g., RT(I, t)), was measured as follows, in two steps:

$$RT(i,t) = (1 - AS(i)) * D(i,t) * (RTmax - DT(i)) + DT(i) + e \qquad \text{(Step 1)}$$

$$RT(i,t) = (1 - AS(i)) * D(i,t) * (RT(i,t) - DT(i)) + DT(i) + e \qquad \text{(Step 2)}$$

Where RT(i, t) is the RT of participant *i* at trial *t*, AS(i) is the measure of specific ability of participant *i*, D(i, t) is the delay time of participant i in trial *t*, *e* is noise, and RTmax corresponds to the expected observed maximum value of RT in a comparable cognitive task (e.g., 20000). The RTmax is used to initialize the simulated measure of RT and is replaced with the actual simulated RT in the second step.

Two types of simulations were completed, which were following either a random or adaptive schema. In the random simulation, 30 random words were assigned to each participant, regardless of the difficulty of the words. On the other hand, in the adaptive schema simulation, the top 30 words with the highest simulated D(i) were assigned exclusively to the participants with the top 15%/20% simulated AS(i). Each type of simulation was repeated three times, and the mean Pearson correlation coefficient was calculated between the ground truth AS(i), DT(i) and D(i) and their respective models' predictions across the three independent runs.

Sample size analysis using simulated data

The adaptive and random simulation analysis was repeated using different sample sizes (from 100 up to 30000), to evaluate the change in model's performance in case of smaller numbers of participants. The mean Pearson correlation coefficient between the ground truths and the model's predictions of AS, DT(i) and D(i) was calculated for each sample size across three independent runs.

IDoCT accurately predicts ground truth measures of AS and DT in simulation studies

The accuracy of IDoCT when extracting measures of specific cognitive abilities (AS), device and visuomotor delays (DT) and trial difficulties (D and DS) was confirmed by applying it to simulated data, where the ground truth was known. The simulated data were generated as if collected from tasks with pseudo-random and adaptive difficulty designs. Specifically, in the former, defined as random simulation, a combination of 30 trials were assigned randomly to each participant. Instead, in the latter, called adaptive simulation, the trials with the highest simulated measure of difficulty D were assigned exclusively to the participants with the highest simulated measure of ability AS. The aim of this simulation was to approximate a staircase task design, where the difficulty of the following trial depends on the performance of participants in the previous one, resulting in the most difficult trials being assigned only to the best performing participants. In both simulation types, the RT per trial was calculated according to the simulated DT, AS and D of the trial of interest, together with a noise

estimate. In total, the data were simulated three times in order to reliably assess how well the model could recover the simulated ground truth measures of AS, DT, D and DS.

IDoCT was run on timecourses from 30000 simulated individuals, where each trial was characterised by an accuracy, RT and condition label. The mean and standard deviation of the Pearson's correlation between the estimated and ground truth measures of AS, DT, D and DS across the different runs of simulations were respectively 0.77 ± 0.03, 0.97 ± 0.00, 0.98 ± 0.00 and 0.99 ± 0.00 for the adaptive simulation analysis, and 0.82 ± 0.01, 0.97±0.00, 0.99±0.00 and 0.99±0.00 for the random simulation (Supplementary Figure 1). The non-ceiling mean correlation of AS was expected as a noise parameter was included when generating the simulated RTs. In the case of D, the model tended to assign difficulty scores within a smaller range compared to the ground truth. However, this was a simple scaling effect, as the trials ranked according to the predicted difficulty scale D followed the same order of those ranked based on the ground truth scale, with the more difficult trials being assigned higher difficulty scores.

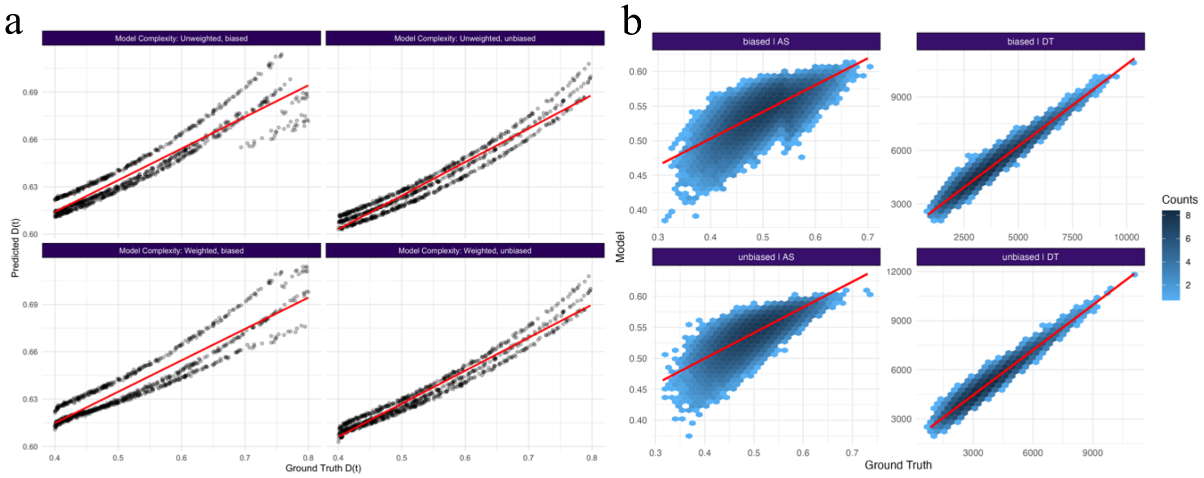

Supplementary Figure 1. Results of analysis of simulated data under adaptive and random task designs. a) Comparison of the predicted vs. ground truth measures of trials difficulty before and after the scaling. b) Comparison of the predicted vs ground truth measures of AS and DT

As intended, in the case of the adaptive simulation analysis, the measures of AS and DT were not affected by the adaptive sampling. However, in case of D, trials that were presented exclusively to participants with higher abilities were originally assigned a lower D score. This bias was effectively corrected when scaling according to the ability of the participants that completed the trials to produce DS.

The model can perform efficiently with as little as 100 samples

To assess the effect of the sample size on the model's performance, the simulation analysis was repeated with different numbers of samples (from 100 up to 30000). The results for both the adaptive and random simulation analysis are reported in Supplementary Figure 4F. When only 100 samples were used to train the model, the minimum r obtained between the predicted and ground truth measures of AS, DT and D were respectively 0.63, 0.96 and 0.83, and the average r was 0.75 ± 0.07, 0.97 ± 0.01, and 0.87 ± 0.03. Therefore, compared to the maximum r achieved across all the simulations conducted (0.85 for AS, 0.99 for DT and 0.99 for D), using 100 samples lead to only a 12%, 2% and 12% drop in average r for respectively AS, DT and D.

However, this drop in performance will be dependent on the dimension of the trials' space, with a more complex trials' space (i.e., higher number of types of trials) requiring a higher number of participants in order for IDoCT to be able to accurately estimates measures of AS, DT, D and DS.

2. IDoCT convergence

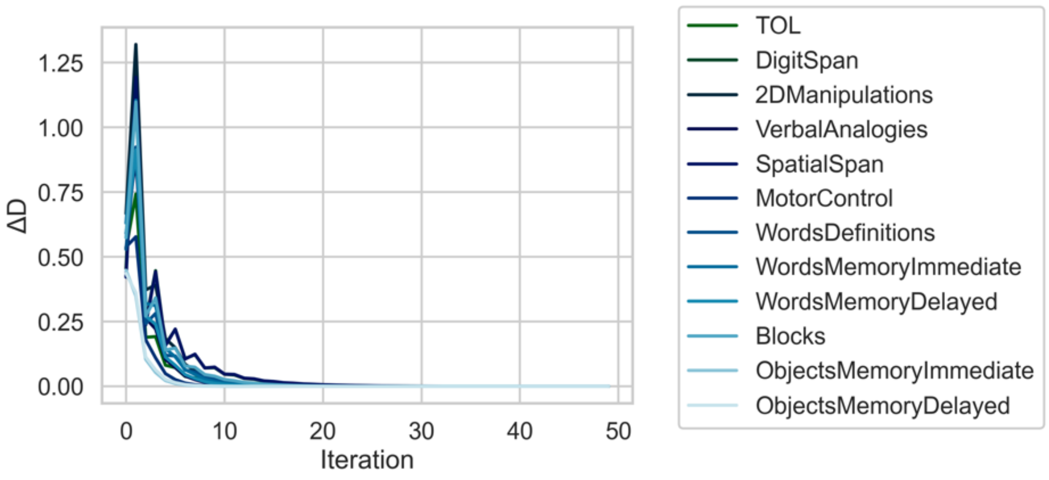

Supplementary Figure 2. IDoCT convergence when estimating trial difficulty D. IDoCT converged by 10 iterations for all cognitive tasks, when the mean change in difficulty D tended to 0.

3. Trial difficulty by task

In the Tower of London spatial planning task, the trials people found most difficult had more convoluted problems, that is, where the same piece must be moved multiple times to achieve the target configuration, and required more moves overall, whereas easier trials required any given piece to be moved just once (Spearman correlation with DS r=0.69 and r=0.71 respectively).

Similarly, in the Blocks spatial planning task (Supplementary Figure 4E), the difficulty of a trial was mainly driven by how convoluted the solution was, in this case, the number of times one block dropped into an empty space when another was removed (correlation r=0.97). The number of moves required to reach the target configuration had a less pronounced relationship to DS (correlation r=0.07).

For the two working memory capacity tasks, Spatial and Digit Span, the more digits or locations that the participants had to remember, the harder was the trial, as shown in Supplementary Figures 3C and 3D (correlation with DS r=0.84 and r=0.92 respectively).

In case of the Words Memory tasks, no significant difference was observed among the words presented (Supplementary Figures 4A and 4B). However, target trials appeared more difficult compared to distractor trials in case of Words Immediate, while the difference was negligible in case of Words Delayed. Instead, a peak in difficulty was evident for the words skirt and blouse for respectively the tasks Words Memory Delayed and Immediate. Since the words are reported in Supplementary Figure 4A and 4B in the order in which they were presented to the participants, this result suggests that the first word shown is the most difficult to recall in case of the Words Immediate task, while the last words presented is the hardest to remember in case of the Delayed Memory task.

In case of the Objects Memory tasks, neither the order in which the different objects were presented, nor the pose appeared to affect the level of difficulty of the trials. However, the category of the objects seemed to play a role, with animal and household objects being consistently more difficulty compared to tools and foods, both in Objects Immediate and Delayed (Supplementary Figure 4C, 4D and 4F).

To assess the word difficulty scale predicted by IDoCT for the Words Definition task, the correlation between the obtained measure of difficulty DS and the frequency of occurrence in the English language of the words included in the cognitive assessment was calculated. As shown in Supplementary Figure 4I, the most frequently used words in English were assigned a lower D and DS compared to the least frequently used ones. A spearman correlation of -0.66 and -0.64 between respectively DS and D with the log frequency measures of the words was found.

For the 2D Manipulations visuospatial reasoning task, difficulty was mainly driven by the location of the answer on the screen, with the answers located along either border being easier to detect compared to those located at the center. The angle of rotation of the target, had a more subtle relationship to difficulty, with a rotation of 180 degrees being associated with slightly easier trials than 90 or 270 degrees (Supplementary Figure 4H).

For Verbal Analogies, analogies with at least one of the sentences from the “Opposite” type group were identified as being generally easier and congruent analogies (e.g. analogies with sentences belonging to the same type) appeared overall easier than incongruent ones. Also, the hardest trials were characterised by pairs of sentences with a semantic distance labelled as far (Supplementary Figure 3E).

Finally, in the Motor Control Task, the further was the location of the current target from the previous location, the higher was the difficulty estimate DS (Spearman correlation between the distance and D and DS 0.91 and 0.87 respectively (Supplementary Figure 3B)).

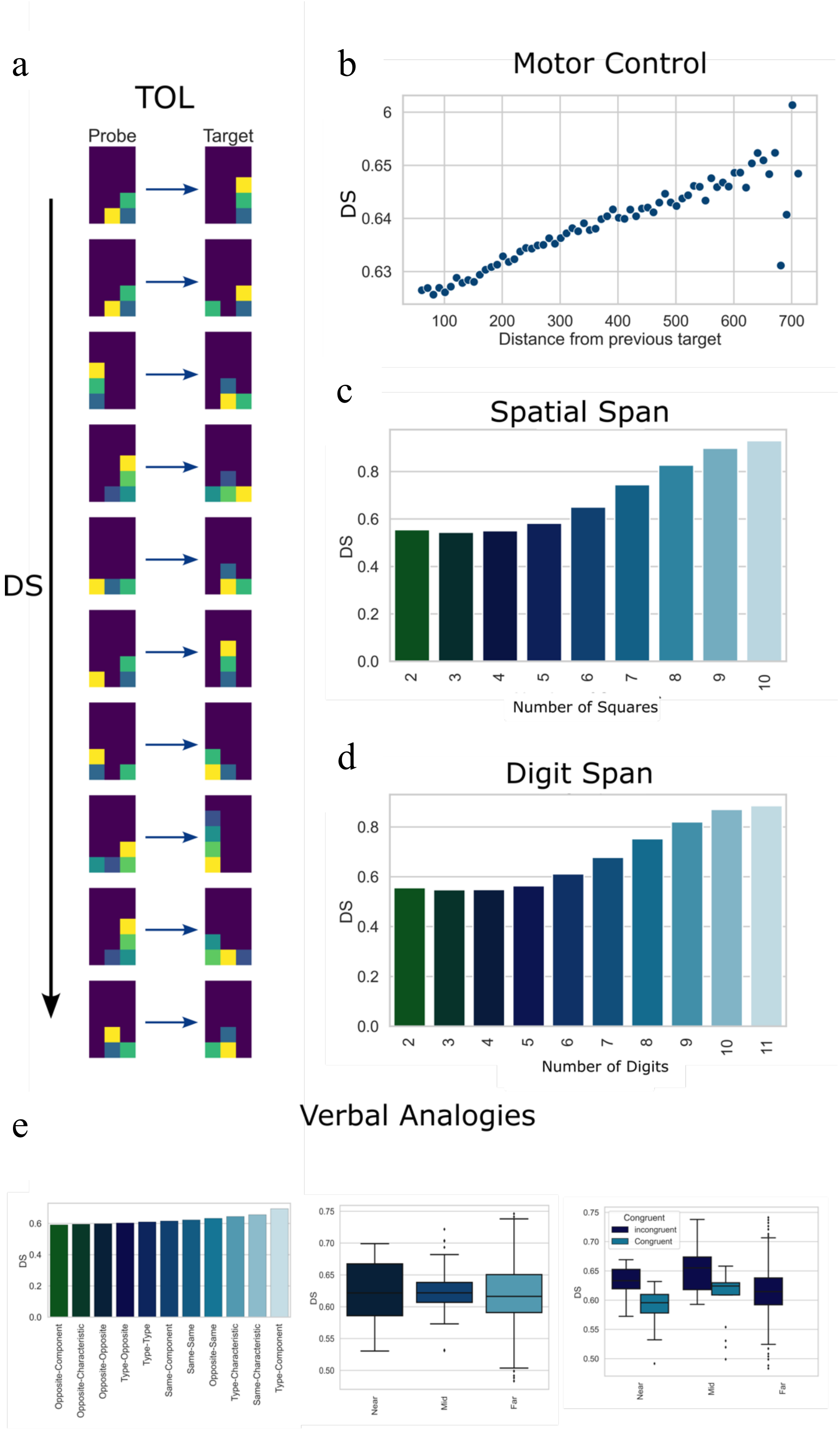

Supplementary Figure 3. Estimated trial difficulty measure DS for TOL, digit span, spatial span and verbal analogy. a) TOL: trials with more convoluted answers and with answers that require a higher number of moves are associated to higher DS scores. b) Motor Control: the father is the target from the previous location, the more difficult is the trial. c) Spatial Span: the more squares participants have to remember, the higher is the DS score. d) Digits Span: the more digits participants have to remember, the higher is the DS score. e) Verbal analogies: analogies with at least one sentence belonging to the "Opposite" group appeared to be easier, whereas incongruent analogies were generally harder.

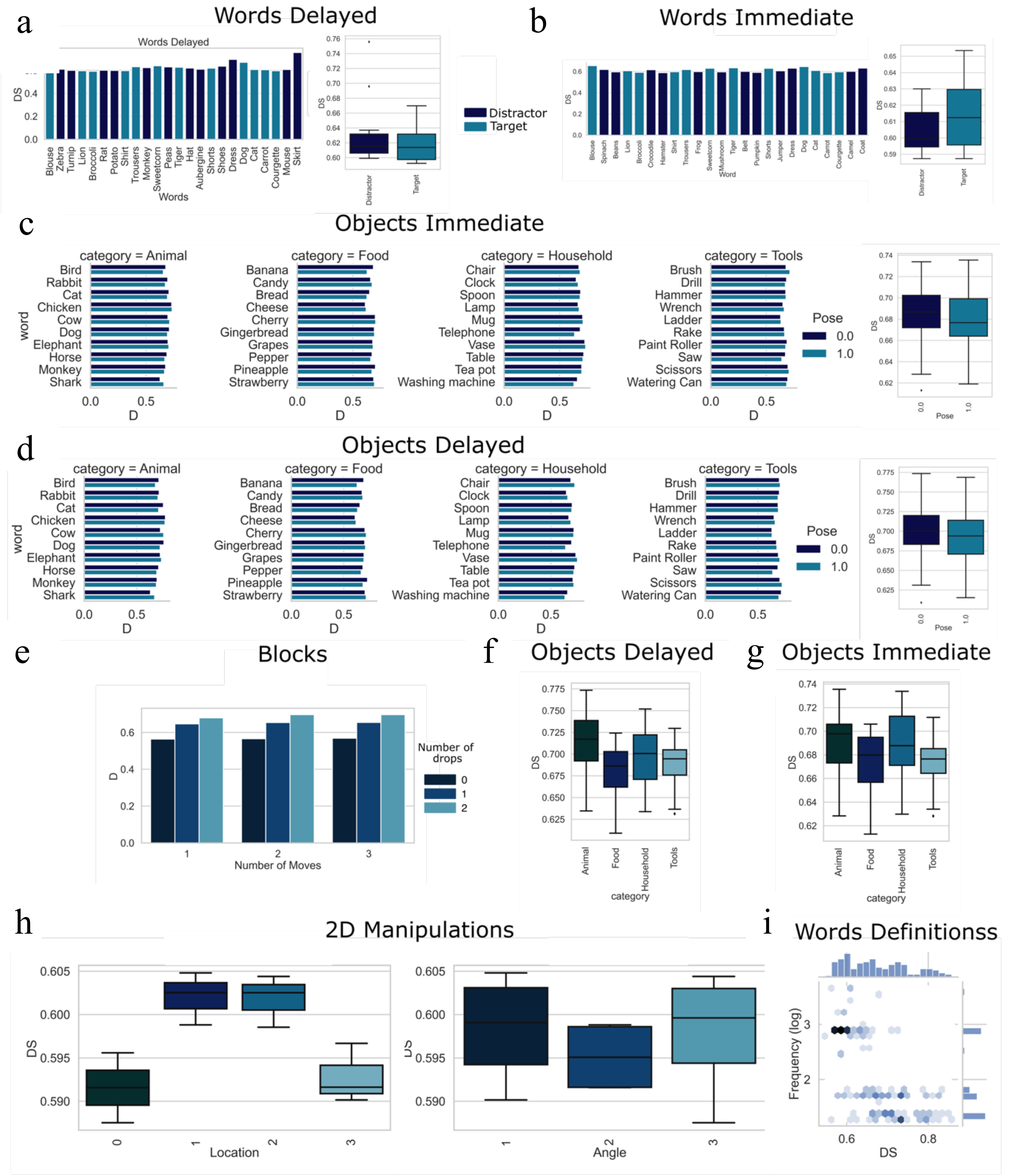

Supplementary Figure 4. Estimated trial difficulty measure DS for Words immediate and delayed, objects immediate and delayed, 2D Manipulations, blocks and Words definition. a) Words delayed: the word presented as last to the participants was the hardest to recall, b) Words immediate: the first word presented to participants was the most difficult to remember and trials with a target word appeared harder compared to those with a distractor word despite the difference in difficulty not being significant, c) Objects Immediate and d) Objects delayed: no significant difference between the poses or words in terms of difficulty, e) Blocks: the more drops were required to reach the desired configuration, the harder was the trial, f) Objects Immediate and g) Objects delayed: objects belonging to the categories "Animal" and "Household" appeared overall harder to remember, and h) 2D Manipulations: the difficulty of a trial was mainly driven by the location of the answer in the screen rather than the angle of rotation, despite grids with a rotation of 180 degrees resulted overall easier, and i) Words definitions: most frequently used words in the English language are associated to easier trials.

4. Correlation with standard metrics

Supplementary Table 1. Correlation between AS, DT, median RT and Number Correct (=Accuracy)

Task	AS – Number Correct	AS - medRT	DT – Number Correct	DT - medRT
**Objects Memory Delayed**	**0.53**	-0.47	-0.11	0.93
**2D Manipulations**	**0.57**	-0.31	-0.84	0.96
**Words Memory Immediate**	**0.85**	-0.45	-0.13	0.89
**Words Memory Delayed**	**0.92**	-0.36	-0.11	0.88
**TOL**	**0.97**	0.02	0.35	0.84
**Verbal Analogies**	**0.69**	-0.24	-0.71	0.95
**Objects Memory Immediate**	**0.48**	-0.62	-0.12	0.92
**Spatial Span**	**0.68**	-0.30	-0.19	0.93
**Digits Span**	**0.57**	-0.24	-0.11	0.94
**Blocks**	**0.98**	-0.27	0.004	0.91
**Motor Control**	**-0.36**	-0.76	0.59	0.97
**Words Definitions**	**0.96**	-0.46	-0.12	0.91

5. Demographics association of median RT and accuracy

A multiple linear regression model was fitted using as outcomes to be predicted the measure of median RT and Accuracy, and as regressors different hot encoded demographics variables (e.g., age in decade, education, and language). Information about ethnicity, sex, residence and handedness were included in the regression model as cofounding factors. The reference categories were 20 years old for age, pre-GCSE for education, and English for language. For each categorical variable, the effect size was measured, which corresponds to the beta coefficient obtained from the regression model divided by the standard deviation of the either median RT or Accuracy. Two separate regression models were fitted for each cognitive task respectively for median RT and Accuracy. The results are presented in Supplementary Table 2 for median RT and Supplementary Table 3 for accuracy.

Supplementary Table 2. Associations with demographics for median RT. The table reports the effect size in SD units.

	2D Manipulations	Blocks	Digits Span	Motor Control	Objects Immediate	Spatial Span	TOL	Verbal Analogies	Words Immediate	Words Definitions	Words Delayed	Objects Delayed
decade10	-0.08	-0.05	0.04	-0.10	-0.11	-0.09	-0.0006	-0.08	-0.06	-0.31	-0.06	-0.15
decade30	0.18	0.13	0.04	0.16	0.01	0.11	0.08	0.21	0.14	0.08	0.19	0.06
decade40	0.37	0.24	0.18	0.30	0.02	0.28	0.16	0.35	0.35	-0.03	0.41	0.12
decade50	0.63	0.46	0.47	0.45	0.07	0.59	0.34	0.59	0.69	-0.09	0.76	0.32
decade60	0.85	0.64	0.73	0.61	0.17	0.82	0.46	0.78	0.99	-0.20	1.07	0.56
decade70	1.10	0.82	1.10	0.80	0.27	1.09	0.66	1.13	1.35	-0.15	1.43	0.77

decade80	1.36	0.98	1.53	0.86	0.62	1.39	0.83	1.58	1.54	-0.13	1.68	1.04
02_Degree	0.02	-0.03	-0.17	0.12	0.05	-0.02	0.18	-0.17	-0.12	-0.49	-0.07	0.009
01_School	-0.01	-0.03	-0.17	0.08	-0.01	-0.04	0.12	-0.09	-0.09	-0.21	-0.05	-0.006
03_PhD	0.009	-0.05	-0.14	0.22	0.06	-0.007	0.17	-0.24	-0.10	-0.73	-0.03	-0.01
Language (Other)	0.15	0.13	0.20	-0.01	0.17	0.15	0.09	0.56	0.18	0.73	0.16	0.19

Supplementary Table 3. Associations with demographics for Accuracy. The table reports the effect size in SD units.

	2D Manipulations	Blocks	Digits Span	Motor Control	Objects Immediate	Spatial Span	TOL	Verbal Analogies	Words Immediate	Words Definitions	Words Delayed	Objects Delayed
decade10	0.09	0.06	-0.19	-0.15	-0.15	-0.07	0.05	0.07	-0.09	-0.17	-0.03	-0.14
decade30	-0.22	-0.11	-0.04	0.08	-0.05	-0.19	0.0002	-0.14	0.01	0.19	-0.06	-0.03
decade40	-0.49	-0.28	-0.06	0.06	-0.10	-0.35	-0.10	-0.23	-0.04	0.37	-0.15	-0.11
decade50	-0.80	-0.51	-0.12	0.06	-0.18	-0.62	-0.26	-0.44	-0.09	0.54	-0.26	-0.18
decade60	-1.06	-0.79	-0.21	0.06	-0.31	-0.87	-0.48	-0.65	-0.22	0.70	-0.38	-0.30
decade70	-1.35	-1.07	-0.38	0.05	-0.45	-1.09	-0.71	-0.94	-0.34	0.76	-0.53	-0.43
decade80	-1.59	-1.34	-0.54	-0.01	-0.62	-1.30	-0.91	-1.27	-0.54	0.76	-0.76	-0.58
02_Degree	0.17	0.39	0.29	0.12	0.30	0.27	0.53	0.61	0.21	0.84	0.21	0.33
01_School	0.13	0.22	0.17	0.11	0.23	0.15	0.31	0.31	0.14	0.44	0.13	0.27
03_PhD	0.23	0.53	0.32	0.15	0.32	0.31	0.65	0.79	0.25	1.11	0.28	0.33
Language (Other)	-0.16	-0.06	0.03	-0.01	-0.04	0.04	-0.09	-0.66	0.16	-0.52	0.18	-0.05

6. Pre-processing of demographics and questionnaire data

The lower tail thresholds applied to the RT distributions of the demographics' features and detected using an automated algorithm are reported in Supplementary Table 4. Since the data were collected across multiple timepoints, the thresholds are timepoint-dependent. When no threshold is reported, it means that the automated algorithm did not detect any sign of improper engagement.

Supplementary Table 4. RT thresholds of demographics features

Demographics	TP1	TP2	TP3	TP4	TP5
**Handedness**	108	6	-	-	-
**Age**	108	1164	-	1703	1717
**Sex**	204	-	-	-	-
**Ethnicity**	237	309	-	-	1086
**Education**	-	205	-	-	957
**Occupation**	199	409	-	-	526
**First Language**	-	793	-	-	-
**Residence**	-	323	-	-	-

The automated algorithm used to detect the RT thresholds for the demographics features consists of the following steps:

- Extraction of RTs different from 0 (RT = 0 means that the question was skipped or not replied to)

- Sorting of RTs
- Identification of the 95% quantile of the RTs distribution
- The 95% quantile is divided by 50 to identify the number of bins to split the RT distribution into. The number 50 can be flexibly modified, the higher is the number, the less stringent is the filtering.
- Starting from the most left bin of the distribution, the height of the bin (which corresponds to the number of people with that range of RTs) is compared to the height of the following bin. If the height of the following bin is lower than the height of the previous one, then the RTs included in the previous bin are marked as unreliable. The process is repeated until the 5% quantile of the RT distribution is reached. The max RT of the last bin marked as unreliable is treated as RT threshold, below which participants were too fast to have properly read the question.
- The RTs below the RT threshold are considered too fast, and the answers of those participants are marked as missing

7. Pre-processing of cognitive data

The lower and upper tail thresholds applied on the RT trial-by-trail distributions of each cognitive task are reported in Supplementary Table 5. The thresholds aimed to detect trials in which participants were either cheating and not properly engaging, or to detect trials characterised by potential software errors at the time of recording. The percentage of observations dropped because of the filtering ranged between 0.2% and 9.1%.

Supplementary Table 5. Trial-by-trial lower and upper tail thresholds of RT distributions for each task

Task	Lower threshold	Upper threshold	Percentage of observations removed (%)
**Objects Memory Delayed**	200	10000	1.4
**2D Manipulations**	200	17500	3.7
**Words Memory Immediate**	200	3000	1.6
**Words Memory Delayed**	200	3000	2.0
**TOL**	1500	40000	9.1
**Verbal Analogies**	200	14000	2.4
**Objects Memory Immediate**	200	10000	5.2
**Spatial Span**	200	5000	0.2
**Digits Span**	200	5000	0.3
**Blocks**	200	20000	1.7
**Motor Control**	200	1500	3.3

In addition, participants that completed the same cognitive task across multiple timepoints were removed from the analysis. The percentage of participants excluded per task is reported in Supplementary Table 6.

Supplementary Table 6. Percentage of participants dropped because of having multiple timepoints

Task	Percentage of participants removed (%)
**Objects Memory Delayed**	2.0
**2D Manipulations**	8.7
**Words Memory Immediate**	3.8
**Words Memory Delayed**	3.6
**TOL**	9.4
**Verbal Analogies**	12.0
**Objects Memory Immediate**	2.3
**Spatial Span**	9.0
**Digits Span**	9.3
**Blocks**	9.6
**Motor Control**	0
**Words Definitions**	8.8
